# Right atrial volume index and right atrial volume predict atrial fibrillation recurrence: A meta-analysis

**DOI:** 10.1371/journal.pone.0315590

**Published:** 2024-12-16

**Authors:** Jia-Ming Han, Qian Xie, Xiu-Ying Song, Yu-lan Ma

**Affiliations:** 1 Medical College of Qinghai University, Xining, China; 2 Qinghai Province Cardiovascular and Cerebrovascular Disease Specialist Hospital, Xining, China; Medical University of Vienna, AUSTRIA

## Abstract

**Background:**

Atrial volume index and atrial volume have recently been identified as predictors of atrial fibrillation (AF) recurrence following electrical cardioversion or radiofrequency ablation. However, most studies have reported the relationship between LAVI/LAV and AF recurrence, whereas there is little information on the relationship between RAVI/RAV and AF recurrence. Therefore, we performed a meta-analysis to assess the relationship between the risk of AF recurrence and RAVI/RAV in patients with AF who underwent electrical cardioversion or radiofrequency ablation.

**Methods:**

CNKI, Wanfang Database, Pubmed, Embase, Cochrane Library, and Web of Science were searched up to October 01, 2024. A meta-analysis of relative risk data from prospective and retrospective cohort studies that reported on the relationship between the risk of AF recurrence and RAVI/RAV in patients with AF after electrical cardioversion or radiofrequency ablation was performed.

**Results:**

The results showed that patients with AF recurrence had a higher mean right atrial volume index (RAVI) compared to patients with no recurrence. After electrical cardioversion or radiofrequency ablation, RAVI can independently predict the recurrence of AF (OR = 1.06, 95%CI (1.02, 1.11)). The average right atrial volume (RAV) of patients with AF recurrence was higher than that of patients without AF recurrence. After electrical cardioversion or radiofrequency ablation, RAV can independently predict the recurrence of AF (OR = 1.02, 95%CI (1.00, 1.05)).

**Conclusion:**

Patients with AF recurrence after electrical cardioversion or radio frequency ablation had higher mean RAVI and RAV compared to patients with no recurrence. After electrical cardioversion or radiofrequency ablation in patients with AF, higher levels of RAVI and RAV increase the chance of recurrence of AF.

## Introduction

Atrial fibrillation (AF) is a common type of rapid arrhythmia. As an increasingly serious public health problem, its incidence is increasing year after year globally [1]. According to statistics, there are as many as 33.5 million AF patients worldwide, and the incidence of AF is increasing as the average population ages [2]. Although current methods for diagnosing and treating AF are constantly being updated and developed, resulting in a decrease in the total mortality rate directly related to AF, but the total hospital stay of AF patients continues to rise. The cause of this phenomenon is not AF itself, but rather its complications [3]. AF is often accompanied by a number of clinical complications, the most common of which are peripheral arterial embolism, sudden cardiac death, and heart failure. These complications have a significant impact on the quality of life and long-term survival of patients with AF [4]. Atrial fibrillation increases the risk of death in the elderly, specifically sudden cardiac death. A retrospective study of older people found that patients with an initial diagnosis of atrial fibrillation had a mortality rate of 19.5% within one year and 48.8% within five years. Another 10-year follow-up study of people aged 55 to 74 found that men with atrial fibrillation died at a rate of 61.5%, while men without atrial fibrillation died at a rate of 30.0% [5]. Currently, medication and ECV treatment are commonly used for symptomatic AF patients. When antiarrhythmic drugs are ineffective, radiofrequency ablation has become the mainstream treatment strategy to delay and treat AF [6, 7]. However, the recurrence rate of AF remained high after restoration of sinus rhythm after electrical cardioversion and radiofrequency ablation treatment. Research has shown that age, gender, structural heart disease, heart failure, obstructive sleep apnea, coronary calcium score, ejection fraction, creatinine, and triglyceride levels all increase the risk of AF recurrence [8–12].

The pathophysiology of AF is complex, involving pro-inflammatory reactions and atrial fibrosis, which result in atrial restructuring and electrophysiological remodeling [13]. Structural atrial remodeling is a process associated with the occurrence, persistence, and development of AF and has been shown to be closely related to AF recurrence [14]. Atrial fibrosis and atrial enlargement have been identified as important factors in the long-term development of AF [15]. Most studies have confirmed the relationship between left atrial factors and AF recurrence, including the degree of left atrial fibrosis, left atrial volume, and left atrial ejection fraction [16–18]. However, there are few reports on the relationship between right atrial-related factors and AF recurrence. Therefore, we performed a systematic review and meta-analysis of published articles to assess the relationship between the risk of AF recurrence and RAVI/RAV in patients with AF undergoing electrical cardioversion or radiofrequency ablation.

## Methods

### Search strategy

We (QX and XS) searched several electronic databases (CNKI, Wanfang Database, Pubmed, Embase, Cochrane Library, and Web of Science) up to 01 October 2024 for prospective cohort studies and retrospective cohort studies, reporting on the relationship between the risk of AF recurrence and RAVI/RAV in patients with AF who underwent electrical cardioversion or radiofrequency ablation. Indexing terms included ((recurrence of atrial fibrillation) AND ((right atrial volume) OR (right atrial volume index)) AND ((electrical cardioversion) OR (radiofrequency ablation))) without language restrictions. At the same time, the references in the retrieved literature are searched for additional information. For some studies lacking key data, we contacted the author via email to supplement the data. Because all analyses were based on previously published studies, no ethical approval or patient consent was required. The study protocol was registered in the PROSPERO database (available from: https://www.crd.york.ac.uk/PROSPERO. ID: CRD42024576462).

### Inclusion criteria

We (QX and XS) included prospective cohort studies and retrospective cohort studies with a study population that included patients with AF after electrical cardioversion or radiofrequency ablation. All titles and abstracts were initially screened to ensure they met the eligibility requirements. All studies must meet the following criteria before they are included in the study: (I) The study subjects were patients with atrial fibrillation following radiofrequency ablation or electrical cardioversion; (II) The study explored the relationship between patients’ RAVI or RAV levels and the risk of atrial fibrillation recurrence, and provided the corresponding HR or OR values.

### Exclusion criteria

Exclusion criteria were listed as follows: (I) Lacked a control group; (II) missing data and data that could not be extracted or calculated from published results; (III) animal experiment studies, commentaries, reviews, or case reports; (IV) any duplicate publication data; (V) low-quality studies.

### Data extraction

Two reviewers (QX and XS) worked independently to extract the data. A third reviewer (YM) was invited to participate in the discussion to reach a consensus. The following information was extracted from each study: the first author’s name, publication data, country, disease status, sample size, average age, sex ratio, proportion of hypertension, proportion of diabetes, proportion of dyslipidemia, proportion of heart failure、surgical method、imaging used、Mean follow-up months、recurrence detection method、number of people with recurrence of AF、number of people without recurrence of AF、mean RAVI and mean RAV.

### Assessment of methodological quality

The Newcastle-Ottawa Quality Assessment Scale (NOS) was used to evaluate the included studies’ quality [19]. The scale assesses the study’s quality based on the sum of three scores: selection, comparability, and outcome. The quality of the included studies was classified as low (scores 1–4), moderate (scores 5–6), or high (scores 7–9) based on the NOS scores. Low-quality studies are excluded from meta-analysis.

### Statistical analysis

For studies that only reported the Hazard ratio, HR was chosen as the best estimate of OR. ORs were logarithmically transformed, and the standard error was calculated using Log OR and the 95% confidence interval (CI). Adjusted ORs were used when individual studies used the Cox proportional hazard model to account for potential confounders. All the data were analyzed by STATA18.0 software, and Q and I^2^ statistics were used to evaluate the heterogeneity of the included study (Low, moderate, and high heterogeneity were defined as I^2^ values of 0–25, 25–49, and >50%, respectively.). Because of statistical heterogeneity among the included literature, a random effects model was used for meta-analysis. To investigate the source of heterogeneity, the following variables were used: publication data, country, sample size, average age, sex ratio, mean follow-up months, mean RAVI, mean RAV, proportion of hypertension, proportion of diabetes. Begg’s and Egger’s tests were used to determine whether there was publication bias, and a sensitivity analysis was performed to test the results’ reliability by eliminating each of the included studies sequentially. Furthermore, when the results revealed publication bias, the impact of publication bias on the results was assessed using the trim and fill method.

## Result

### Characteristics of the included studies

**Fig 1:**
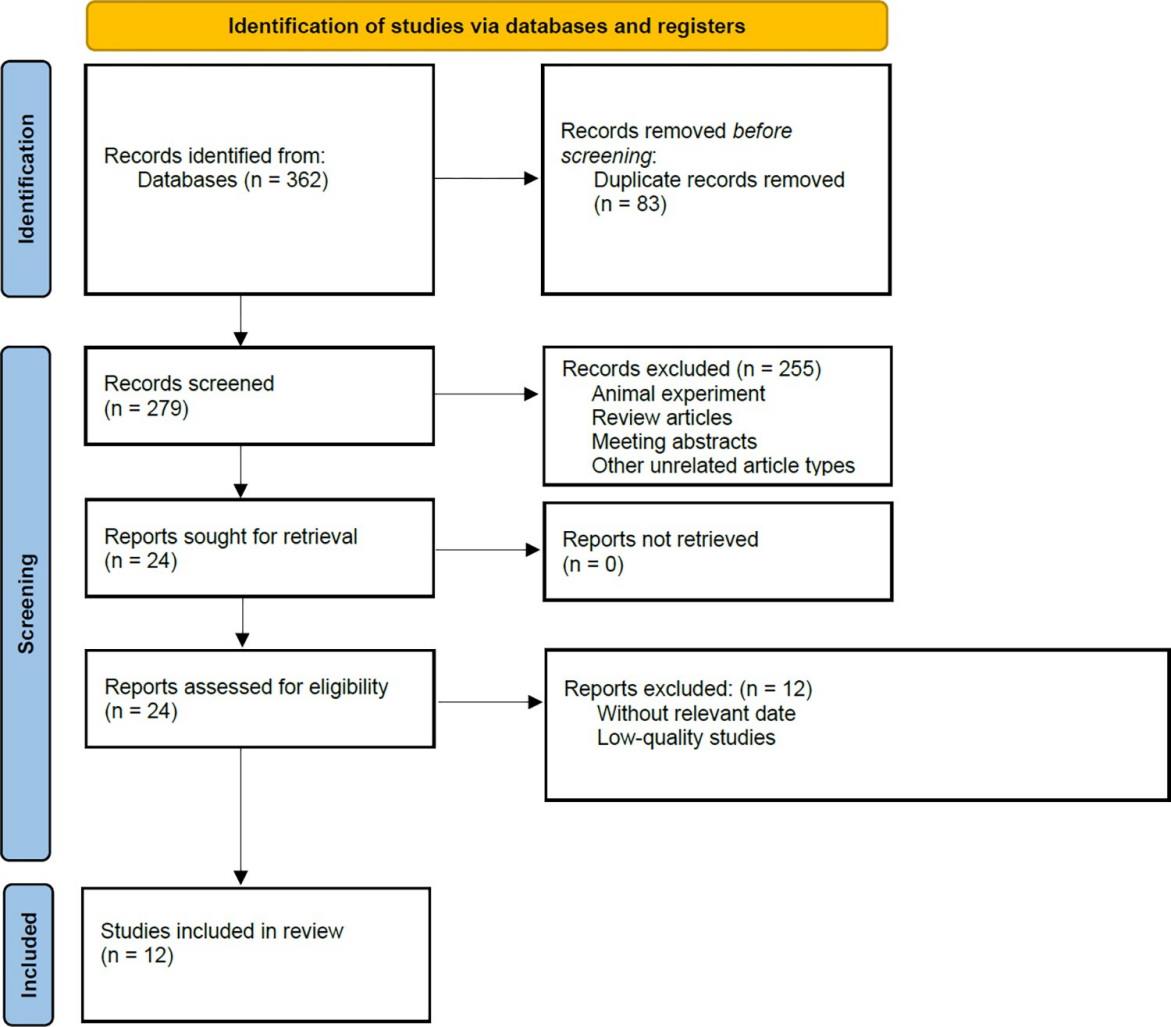
Flowchart depicting the database search and study inclusion processes.

A total of 362 potentially relevant publications were identified, including CNKI (n = 68), WanFang database (n = 8), Embase (n = 98), PubMed (n = 86), Web of science (n = 95), Cochrane library (n = 7). After screening the titles and abstracts, our meta-analysis included 12 studies with a total of 1628 participants [20–31]. The flow chart for the study selection is shown in Fig 1.

Six of the twelve articles included in the meta-analysis examined the relationship between the risk of AF recurrence and RAVI levels in patients who had undergone electrical cardioversion or radiofrequency ablation [20–25]. The average age of the participants in the included studies ranged from 56.9 to 63 years, the proportion of men ranged from 60.2 to 82.4%, and the RAVI ranged from 44.84 to 99.9 ml/m^2^. Because one of the studies evaluated the relationship between AF recurrence risk and RAVI levels based on different follow-up times, it was divided and included in the analysis [23]. Three of the studies, which assessed the relationship between the risk of AF recurrence and RAVI levels based on different AF types, were included separately in the analysis [20, 24, 25].

Seven of the twelve articles included in the meta-analysis examined the relationship between the risk of AF recurrence and RAV levels in patients who had undergone electrical cardioversion or radiofrequency ablation [21, 26–31]. The average age of the participants in the included studies ranged from 59 to 72 years, the proportion of men ranged from 55 to 81.5%, and the RAV ranged from 35 to 126.8 ml.

Nine of the included studies were prospective cohort studies, while three were retrospective cohort studies. These studies had an average follow-up time of 3 to 23.6 months and assessed RAV and RAVI with CT, CMR, and Echocardiography. During the follow-up period, patients were contacted through phone and outpatient visits. All studies used Holter to monitor and diagnose AF recurrence. The study quality results revealed that one study had NOS scores of six [26], three studies had NOS scores of seven [20, 23, 27], two studies had NOS scores of eight [21, 30], and six had a NOS score of nine [22, 24, 25, 28, 29, 31]. The general characteristics of the studies included in this review are shown in S1–S4 Tables.

### Meta-analysis of the difference in right atrial volume index between patients with and without atrial fibrillation recurrence after electrical cardioversion or radiofrequency ablation

**Fig 2:**
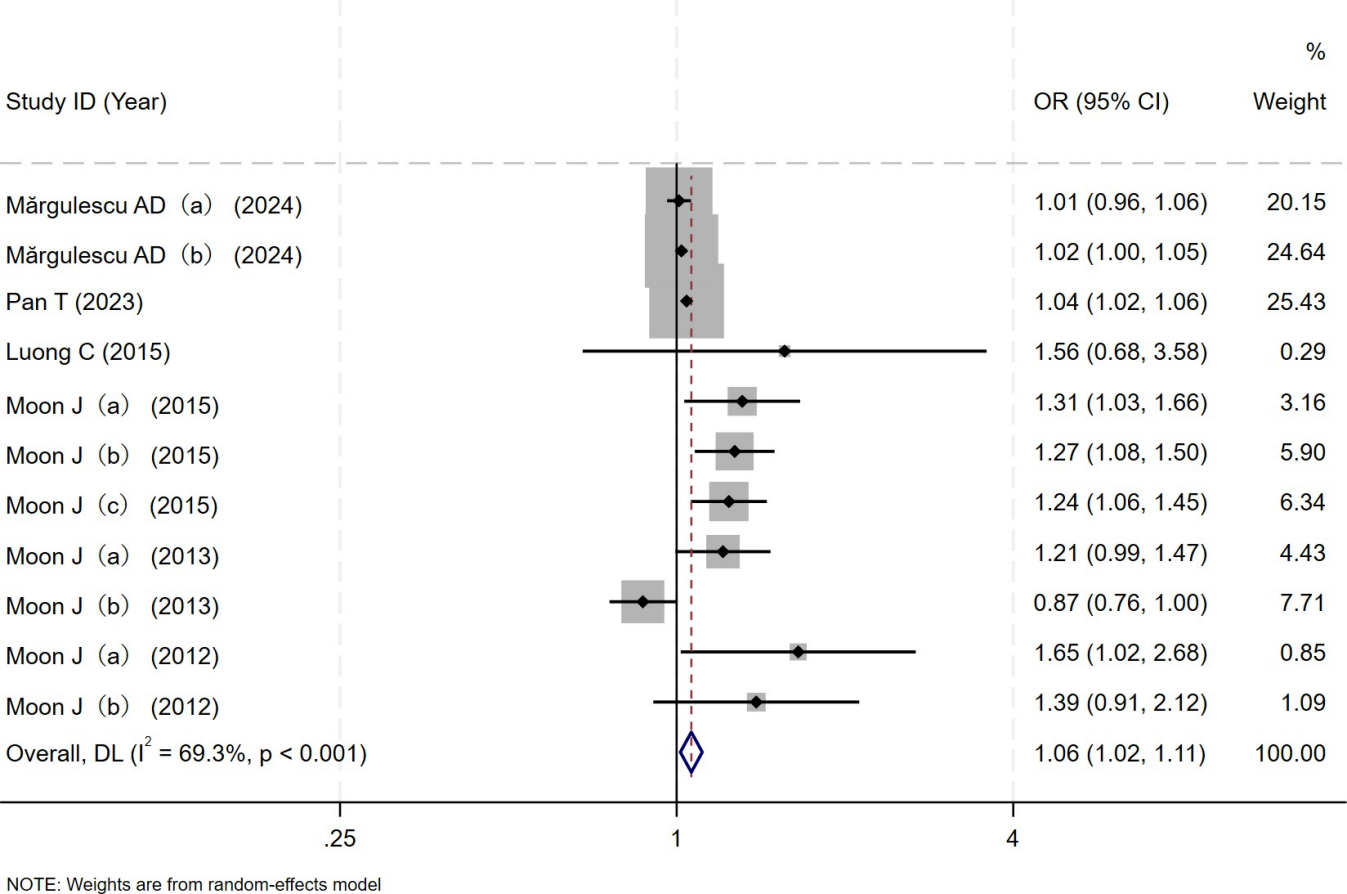
Forest plot of the relationship between the risk of atrial fibrillation recurrence and RAVI levels in patients with atrial fibrillation who underwent electrical cardioversion or radiofrequency ablation.

The relationship between the risk of AF recurrence and RAVI levels in patients with AF who underwent electrical cardioversion or radiofrequency ablation was reported in six of the included studies. S1 and S2 Tables summarize the characteristics of the included studies. A random effect model was used for Meta analysis due to the statistical heterogeneity among the studies (I^2^ = 69.3% P<0.001). The results revealed that RAVI levels were associated with the risk of AF recurrence in patients with AF who underwent electrical cardioversion or radiofrequency ablation (OR = 1.06, 95%CI (1.02, 1.11); Fig 2). The higher the right atrial volume index, the greater the risk of AF recurrence.

Subgroup analysis is required to identify the source of heterogeneity in studies on the relationship between the risk of AF recurrence and RAVI levels in patients with AF due to statistical heterogeneity (I^2^ = 69.3% P<0.001). To investigate the source of heterogeneity, the following variables were used: publication data, country, sample size, average age, sex ratio, Mean follow-up months, mean RAVI, proportion of hypertension, proportion of diabetes, proportion of dyslipidemia and proportion of heart failure. Following the subgroup analysis, the results revealed that no significant source of heterogeneity was discovered, indicating that additional sensitivity analysis is required (S5 Table).

**Fig 3:**
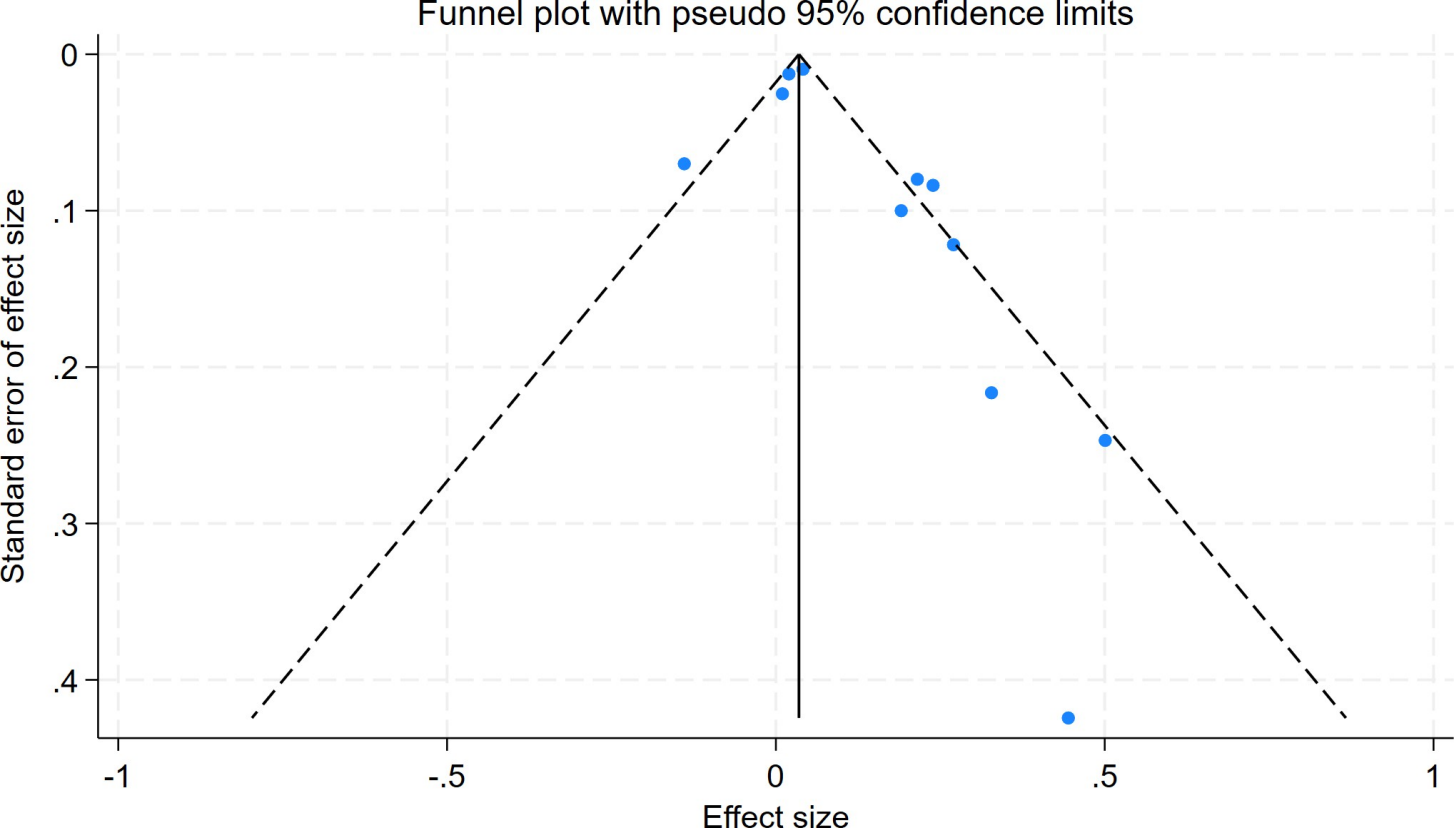
Funnel diagram of the relationship between the risk of atrial fibrillation recurrence and RAVI levels in patients with atrial fibrillation who underwent electrical cardioversion or radiofrequency ablation.

**Fig 4:**
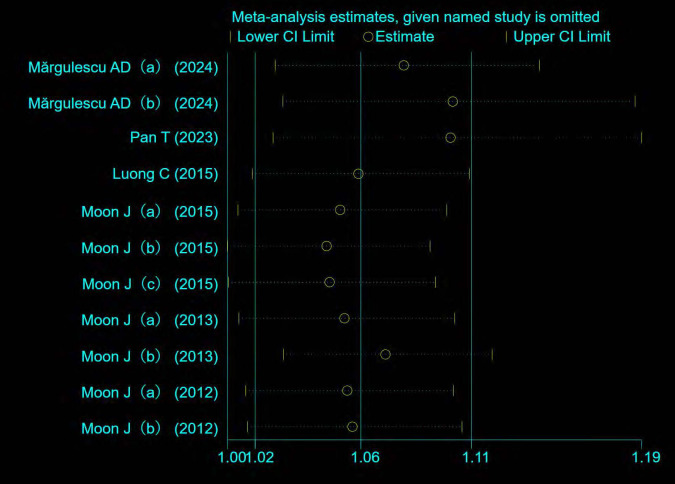
Sensitivity analysis of the relationship between the risk of atrial fibrillation recurrence and RAVI levels in patients with atrial fibrillation who underwent electrical cardioversion or radiofrequency ablation.

**Fig 5:**
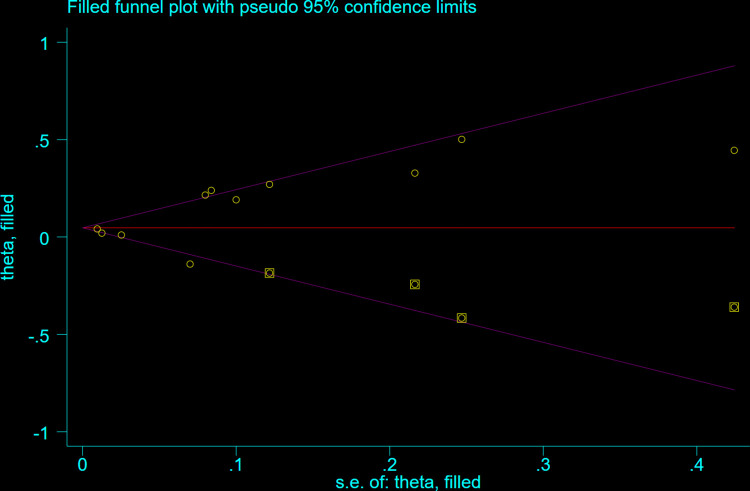
Trim and fill sensitivity analyses of the relationship between the risk of atrial fibrillation recurrence and RAVI levels in patients with atrial fibrillation who underwent electrical cardioversion or radiofrequency ablation.

The Egger test (t = 2.06, P = 0.070) and Begg test (Z = 0.78, P = 0.436) indicated no statistically significant publication bias in this meta-analysis. The funnel diagram depicts a roughly symmetrical distribution (Fig 3). The sensitivity analysis revealed that the consolidated results were all stable (Fig 4). And the sensitivity analyses of Trim and Fill Method showed the result was reliable (After including four virtual studies, the combined results remained unchanged; Fig 5).

### Meta-analysis of the difference in right atrial volume between patients with and without atrial fibrillation recurrence after electrical cardioversion or radiofrequency ablation

**Fig 6:**
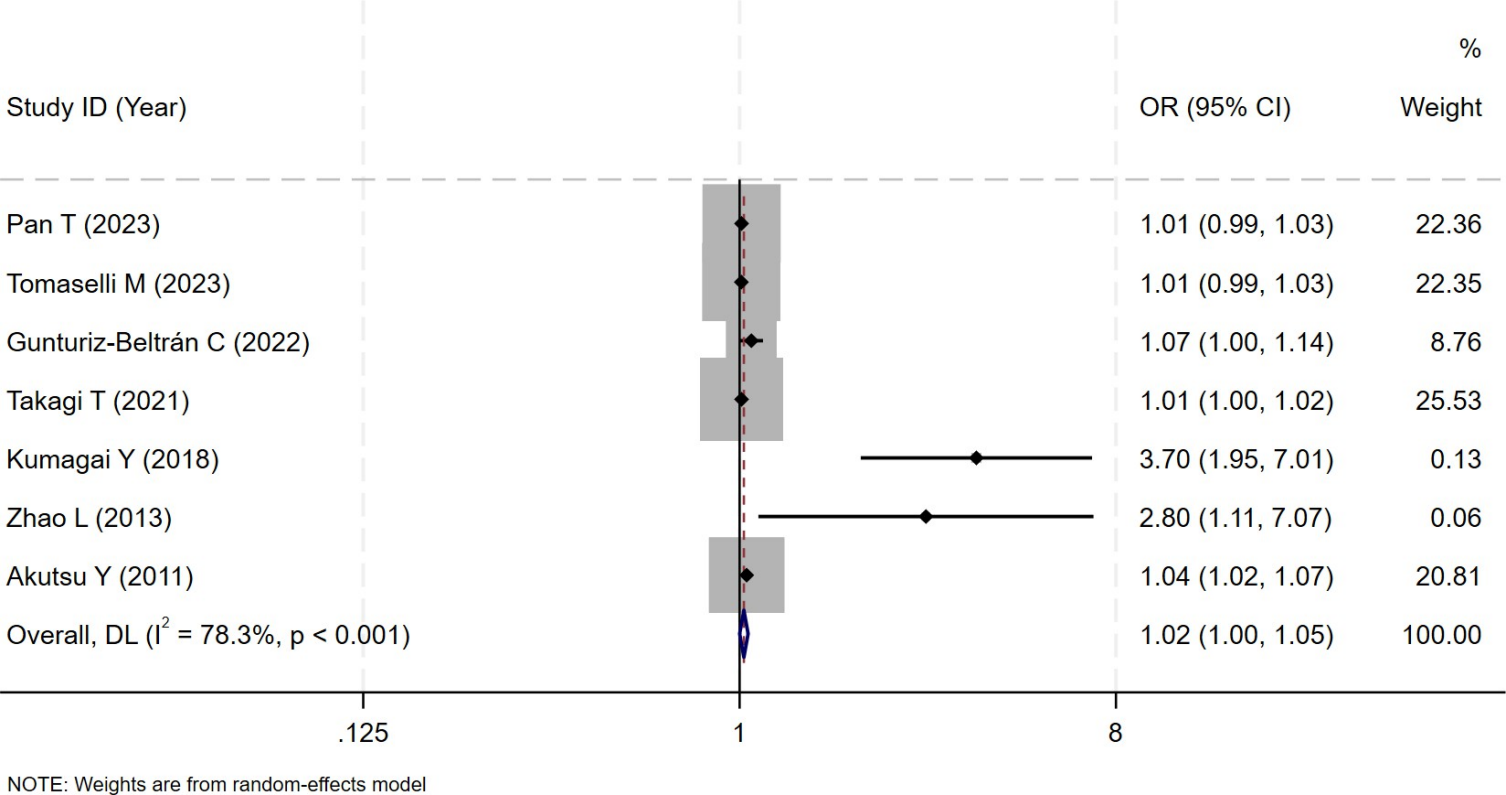
Forest plot of the relationship between the risk of atrial fibrillation recurrence and RAV levels in patients with atrial fibrillation who underwent electrical cardioversion or radiofrequency ablation.

The relationship between the risk of AF recurrence and RAV levels in patients with AF who underwent electrical cardioversion or radiofrequency ablation was reported in seven of the included studies. S3 and S4 Tables summarize the characteristics of the included studies. A random effect model was used for Meta analysis due to the statistical heterogeneity among the studies (I^2^ = 78.3%, P<0.001). The results revealed that RAV levels were associated with the risk of AF recurrence in patients with AF who underwent electrical cardioversion or radiofrequency ablation (OR = 1.02, 95%CI (1.00, 1.05); Fig 6). The higher the right atrial volume, the greater the risk of AF recurrence.

Subgroup analysis is required to identify the source of heterogeneity in studies on the relationship between the risk of AF recurrence and RAV levels in patients with AF due to statistical heterogeneity (I^2^ = 78.3%, P<0.001). To investigate the source of heterogeneity, the following variables were used: publication data, country, sample size, average age, sex ratio, mean follow-up months, mean RAV, proportion of hypertension, proportion of diabetes. Following the subgroup analysis, the results revealed that no significant source of heterogeneity was discovered, indicating that additional sensitivity analysis is required (S6 Table).

**Fig 7:**
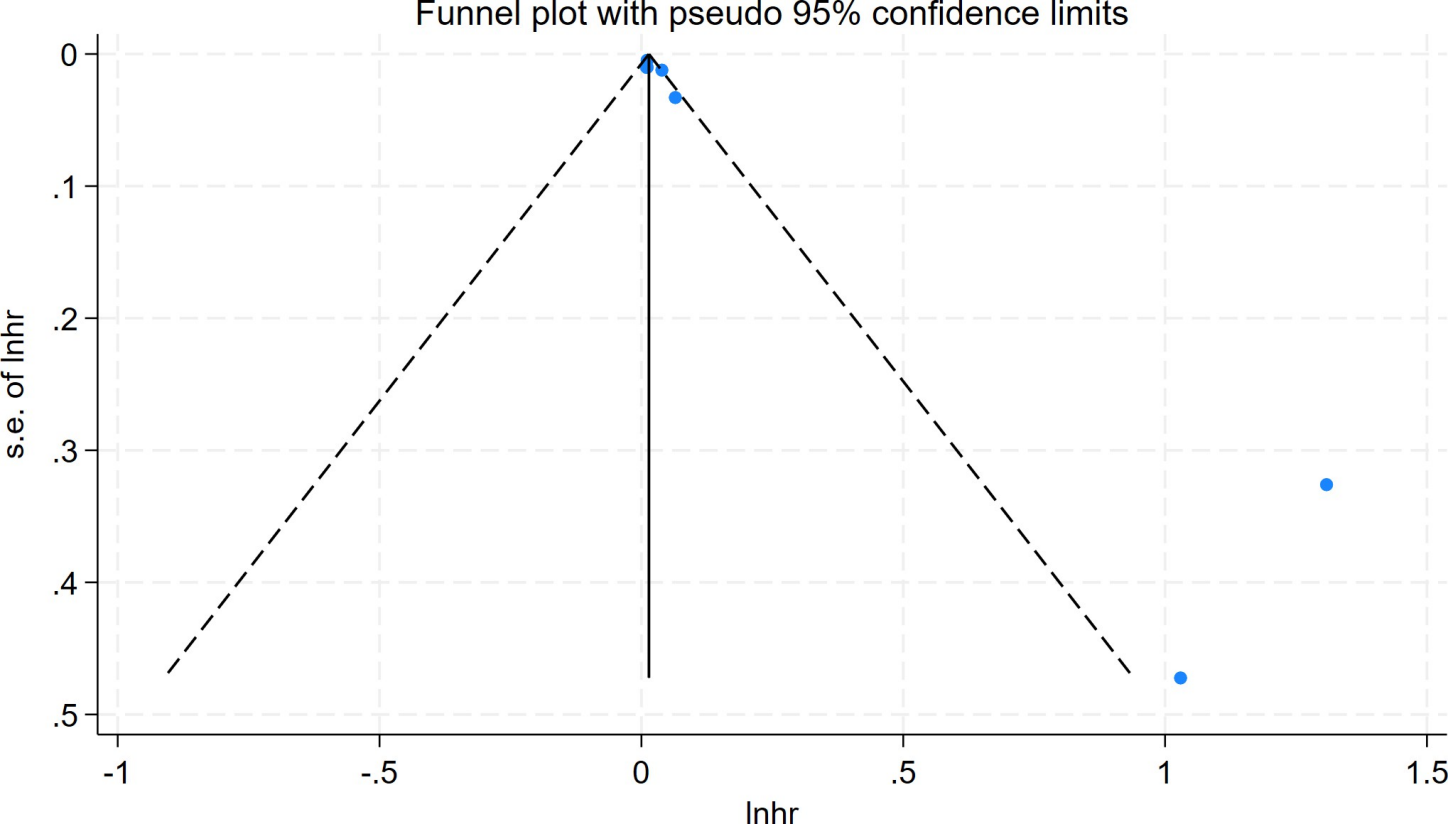
Funnel diagram of the relationship between the risk of atrial fibrillation recurrence and RAV levels in patients with atrial fibrillation who underwent electrical cardioversion or radiofrequency ablation.

**Fig 8:**
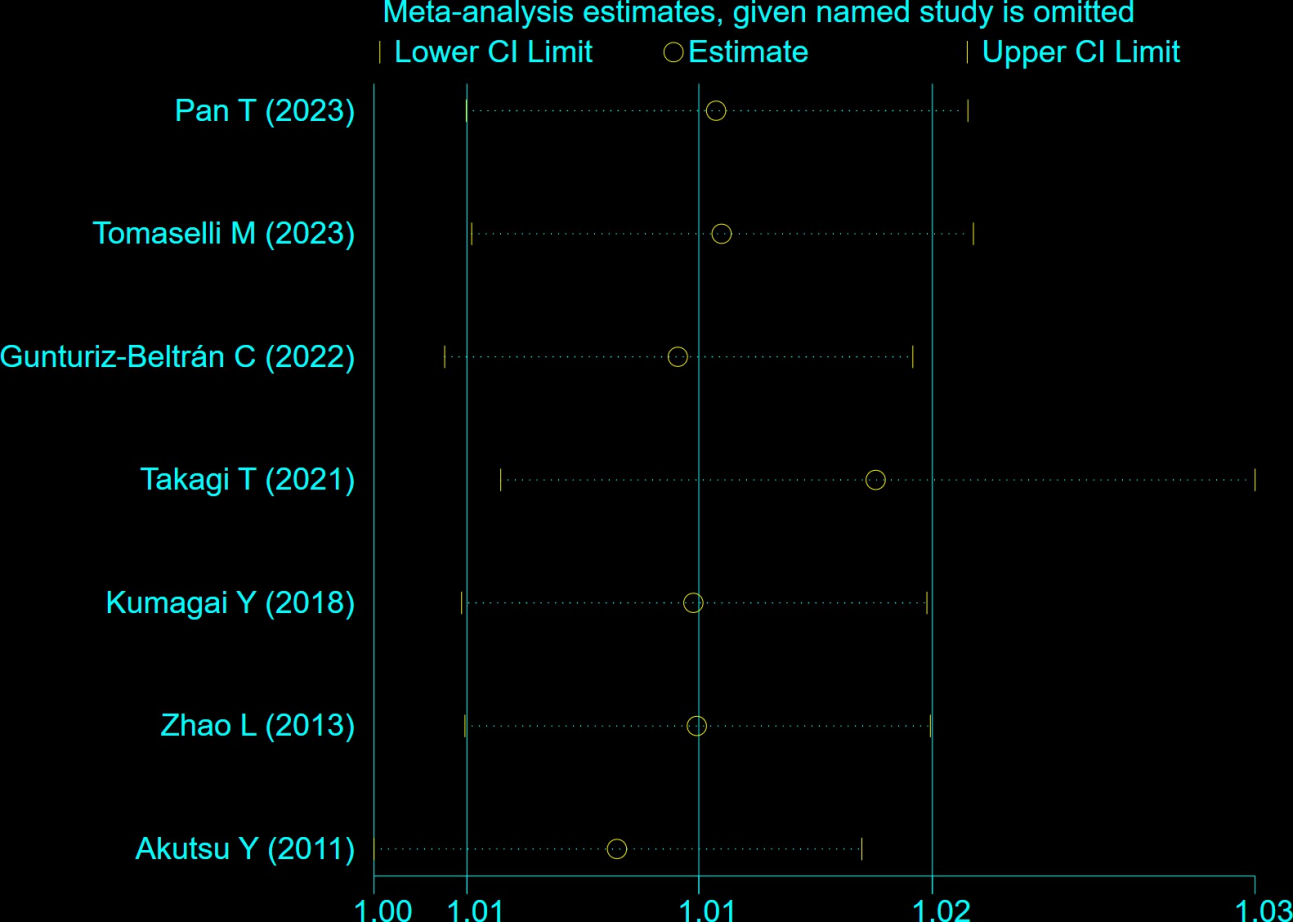
Sensitivity analysis of the relationship between the risk of atrial fibrillation recurrence and RAV levels in patients with atrial fibrillation who underwent electrical cardioversion or radiofrequency ablation.

**Fig 9:**
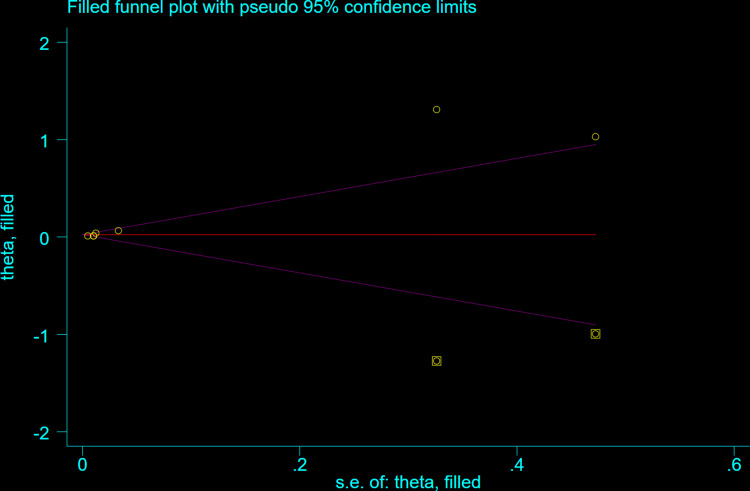
Trim and fill sensitivity analyses of the relationship between the risk of atrial fibrillation recurrence and RAV levels in patients with atrial fibrillation who underwent electrical cardioversion or radiofrequency ablation.

The Egger test (t = 4.12, P = 0.009) and Begg test (Z = 2.10, P = 0.035) indicated that there may be a publication bias in this meta-analysis. The funnel diagram depicts a roughly symmetrical distribution (Fig 7). Despite the presence of publication bias, the sensitivity analysis revealed that the consolidated results were stable (Fig 8). And the sensitivity analyses of Trim and Fill Method showed the result was reliable (After including two virtual studies, the combined results remained unchanged; Fig 9).

## Discussion

The results of this study suggest that there may be a link between the risk of recurrent atrial fibrillation and RAVI/RAV in patients who have had atrial fibrillation treated with radiofrequency ablation or electric cardioversion. In patients with AF, high levels of RAVI and RAV increase the risk of recurrence compared to low levels of RAVI and RAV.

Atrial remodeling, which is characterized by changes in atrial structure and function, is central to the persistence and progression of AF. Atrial remodeling is caused by a combination of factors including inflammation, oxidative stress, increased sympathetic nerve activity, and activation of the renin-angiotensin-aldosterone system [32–34]. During the process of atrial remodeling, the formation of myocardial fibrosis is one of the important factors leading to the development of AF. Atrial fibrosis slows the conduction of local areas, which affects the electrical signal conduction of the heart, resulting in arrhythmia [35]. Patients with AF frequently exhibit fibrotic remodeling of both atria [36]. With the occurrence and development of AF patients, the cardiomyocytes in their atria continue to decrease and be replaced by fibrous tissue, exacerbating the changes in the atrial structure of AF patients and making AF easier to trigger and maintain. Recent research has found that patients with paroxysmal AF have significantly more interstitial fibrosis in the right atrial appendage than healthy people [37]. Meanwhile, Patients with permanent AF had larger atria and a more impaired atrial reservoir strain than patients with paroxysmal AF [38]. Currently, the relationship between left atrial remodeling and AF has been extensively researched and confirmed. Recently, the structural change of the right atrium has been confirmed to be one of the risk factors causing AF in the normal population, and a higher RA volume index is independently correlated with the incidence of AF [39]. Furthermore, the frequency of AF in patients with paroxysmal AF is related to the right heart structure and RV deformation properties of patients, and the more severely the right heart structure and RV deformation properties are damaged, the higher the frequency of AF [40]. In addition, older patients with persistent or permanent AF are more likely to have right atrial enlargement, which increases the risk of heart failure, systemic embolism, and death in AF patients [41].

After converting AF patients to sinus rhythm with electrical cardioversion or radiofrequency ablation, the risk of AF recurrence remains high. Therefore, long-term treatment and health management are critical for lowering the risk of AF recurrence. Extensive evidence shows that large right atrial volume causes a vicious cycle of atrial remodeling and AF [42]. For AF patients after radiofrequency ablation, the size of the right atrial volume is significantly related to the risk of AF recurrence, and patients with AF recurrence have a significantly larger average right atrial diameter than patients without recurrence [43, 44]. Patients with long-term persistent AF after CPVI treatment and RAVI/LAVI > 100.1 were more likely to have postoperative arrhythmia recurrence [45]. For AF patients after electrical cardioversion, RAVI was demonstrated to be an independent predictor of AF recurrence, and it was superior to LAVI in predicting AF recurrence at 6 months after electrical cardioversion [22]. In addition, the indexed right atrial area was more predictive of AF recurrence after electrical cardioversion than LAVI [46].

The studies discussed above show a link between the risk of AF recurrence and RAVI/RAV after electrical cardioversion or radiofrequency ablation in patients with AF, and provided preliminary evidence for the prediction of RAVI/RAV for the risk of AF recurrence. However, as AF patients frequently have multiple diseases and different drug use histories, it is impossible to determine whether high levels of RAVI and RAV are a direct cause of AF recurrence. As a result, more research is needed to investigate the link between the risk of AF recurrence and RAVI/RAV after electrical cardioversion or radiofrequency ablation in patients with AF.

## Study limitations

When interpreting the results, many potential limitations of our study must be considered. First, there are some differences between research groups, such as research objects, countries, regions, races, and eating habits, which may cause results to differ. Second, for the study of RAVI and RAV, there is no systematic correction of various factors of the research object, and the results may be influenced by multiple factors. Third, these studies had varying proportions of patients with paroxysmal, persistent, and permanent AF. We were unable to conduct further subgroup analysis due to a lack of relevant data. Fourth, the exclusion of some literatures that lack relevant data or are of poor quality may have an impact on the final results.

## Conclusions

The results of this study revealed that patients with recurrent AF following electrical cardioversion or radiofrequency ablation had higher mean RAVI and RAV than patients with AF who did not recurrence. After electrical cardioversion or radiofrequency ablation in patients with AF, higher levels of RAVI and RAV increase the chance of recurrence of AF. We believe that RAVI and RAV can be used as risk assessment indicators of atrial fibrillation recurrence, and that when combined with other prognostic indicators of atrial fibrillation recurrence, they can assist patients in lowering their risk of atrial fibrillation recurrent.

## Supporting information

S1 Table Characteristics of included studies (RVAI). (DOCX)

S2 Table Characteristics of included studies (RVAI). (DOCX)

S3 Table Characteristics of included studies (RVA). (DOCX)

S4 Table Characteristics of included studies (RVA). (DOCX)

S5 Table Subgroup analysis of the relationship between the risk of atrial fibrillation recurrence and RAVI levels in patients with atrial fibrillation who underwent electrical cardioversion or radiofrequency ablation. (DOCX)

S6 Table Subgroup analysis of the relationship between the risk of atrial fibrillation recurrence and RAV levels in patients with atrial fibrillation who underwent electrical cardioversion or radiofrequency ablation. (DOCX)

S7 Table PRISMA 2020 checklist. (DOCX)

S8 Table A numbered table of all studies identified in the literature search. (DOCX)

S9 Table Study quality evaluation via the Newcastle-Ottawa scale. (DOCX)
