# Supplementary material for: Right atrial volume index and right atrial volume predict atrial fibrillation recurrence: A meta-analysis
Source: PLoS One. 2024 Dec 16;19(12):e0315590. doi: 10.1371/journal.pone.0315590 (PMC11649108; doi:10.1371/journal.pone.0315590)
Supplement: S1 Table — (DOCX) [file pone.0315590.s001.docx]

| **Table S1.** Characteristics of included studies（RVAI） | | | | | | | | | | | |  |  |
| --- | --- | --- | --- | --- | --- | --- | --- | --- | --- | --- | --- | --- | --- |
| Study | Year | Country | Disease status | Sample | Age (y) | Male，% | Hypertension，% | Diabetes，% | Dyslipidemia，% | Heart Failure，% | |  |  |
|  |  |  |  |  |  |  |  |  |  |  |  |  | |
| Mărgulescu AD ^[20]^ | 2024 | Spain | PaAF and PeAF | 85 | 56.9±9.1 | 82.4 | 47.1 | N/A | N/A | N/A | |  | |
|  |  |  |  |  |  |  |  |  |  |  |  |  | |
| Pan T ^[21]^ | 2023 | China | PaAF and PeAF | 297 | 61.1±11 | 60.2 | 55.6 | 17.1 | 22.9 | 23.9 | |  | |
|  |  |  |  |  |  |  |  |  |  |  |  |  | |
| Luong C ^[22]^ | 2015 | Canada | PeAF | 95 | 63±12 | 67.4 | 63 | 15 | 47.4 | 28 | |  | |
|  |  |  |  |  |  |  |  |  |  |  |  |  | |
| Moon J ^[23]^ | 2015 | Republic of Korea | PaAF and PeAF | 111 | 57±11 | 78 | 38 | 5 | 12 | 4 | |  | |
|  |  |  |  |  |  |  |  |  |  |  |  |  | |
| Moon J ^[24]^ | 2013 | Republic of Korea | PaAF and PeAF | 159 | 57±11 | 82 | 39.6 | 5.7 | 11.9 | 3 | |  | |
|  |  |  |  |  |  |  |  |  |  |  |  |  | |
| Moon J ^[25]^ | 2012 | Republic of Korea | PaAF and PeAF | 63 | 57±10 | 74.6 | 49.2 | 3.2 | 11.1 | 1.6 | |  | |
|  |  |  |  |  |  |  |  |  |  |  |  |  | |
| AF，atrial fibrillation；PaAF，Paroxysmal atrial fibrillation；PeAF，Persistent atrial fibrillation. | | | | | | | | | | | |  | |
