# Supplementary material for: Right atrial volume index and right atrial volume predict atrial fibrillation recurrence: A meta-analysis
Source: PLoS One. 2024 Dec 16;19(12):e0315590. doi: 10.1371/journal.pone.0315590 (PMC11649108; doi:10.1371/journal.pone.0315590)
Supplement: S2 Table — (DOCX) [file pone.0315590.s002.docx]

| **S2 Table.** Characteristics of included studies （RVAI） | | | | | | | | | | | | | |  |
| --- | --- | --- | --- | --- | --- | --- | --- | --- | --- | --- | --- | --- | --- | --- |
| Study | Year | Disease status | Surgical method | Imaging used | Mean follow-up months | Recurrence detection method | Number of people with recurrence of atrial fibrillation，n | Number of people without recurrence of atrial fibrillation，n | Mean RAVI，ml/m2 | Mean RAVI，ml/m2 | | HR/OR | NOS |  |
|  |  |  |  |  |  |  |  |  |  | Recurrence | No Recurrence |  |  |  |
| Mărgulescu AD（a）^[20]^ | 2024 | PaAF | PVI | CMR | 11 | Holter | 17 | 28 | 45.9±10.7 | 46.7 ± 11.9 | 45.4 ± 10 | 1.01（0.96，1.06） | 7 |  |
|  |  |  |  |  |  |  |  |  |  |  |  |  |  |  |
| Mărgulescu AD（b）^[20]^ | 2024 | PeAF | PVI | CMR | 11 | Holter | 18 | 22 | 58.5 ± 19.2 | 64.2 ± 22.5 | 53.8 ± 15 | 1.02（0.99，1.04） | 7 |  |
|  |  |  |  |  |  |  |  |  |  |  |  |  |  |  |
| Pan T ^[21]^ | 2023 | PaAF and PeAF | RFA | CT | 12 | Holter and ECG | 83 | 214 | 44.84 | 47.69 | 41.99 | 1.042（1.023，1.062） | 8 |  |
|  |  |  |  |  |  |  |  |  |  |  |  |  |  |  |
| Luong C ^[22]^ | 2015 | PeAF | DCCV | echocardiography | 6 | ECG | 53 | 29 | 45.18 ± 12.1 | 48.5 ± 14.2 | 39.1 ± 6.6 | 1.56（0.497，2.623） | 9 |  |
|  |  |  |  |  |  |  |  |  |  |  |  |  |  |  |
| Moon J（a）^[23]^ | 2015 | PaAF and PeAF | RFCA | MDCT | 3 | Holter and ECG | 26 | 85 | 99.9 ± 24.8 | 107 ± 33 | 86 ± 20 | 1.31 （1.03，1.66） | 7 |  |
|  |  |  |  |  |  |  |  |  |  |  |  |  |  |  |
| Moon J（b）^[23]^ | 2015 | PaAF and PeAF | RFCA | MDCT | 6 | Holter and ECG | 30 | 81 | 99.4 ± 24.6 | 105 ± 31 | 85 ± 20 | 1.27（1.08，1.50） | 7 |  |
|  |  |  |  |  |  |  |  |  |  |  |  |  |  |  |
| Moon J（c）^[23]^ | 2015 | PaAF and PeAF | RFCA | MDCT | 12 | Holter and ECG | 36 | 75 | 92 ± 27 | 103 ± 29 | 85 ± 21 | 1.24（1.06，1.45） | 7 |  |
|  |  |  |  |  |  |  |  |  |  |  |  |  |  |  |
| Moon J（a）^[24]^ | 2013 | PaAF | RFA | MDCT | 12 | Holter and ECG | 25 | 134 | 82.3 ± 23 | 89 ± 23 | 81 ± 23 | 1.21（1.00，1.48） | 9 |  |
|  |  |  |  |  |  |  |  |  |  |  |  |  |  |  |
| Moon J（b）^[24]^ | 2013 | PeAF | RFA | MDCT | 12 | Holter and ECG | 41 | 42 | 96 ± 29.7 | 94 ± 26 | 98 ± 33 | 0.87（0.76，1.00） | 9 |  |
|  |  |  |  |  |  |  |  |  |  |  |  |  |  |  |
| Moon J（a）^[25]^ | 2012 | PeAF | RFA | MDCT | 3 | Holter and ECG | 8 | 15 | 81.3 ± 21.8 | 98 ± 21 | 77 ± 22 | 1.65（1.017，2.677） | 9 |  |
|  |  |  |  |  |  |  |  |  |  |  |  |  |  |  |
| Moon J（b）^[25]^ | 2012 | PeAF | RFA | MDCT | 12 | Holter and ECG | 12 | 11 | 81.2 ± 22.3 | 91 ± 27 | 77 ± 20 | 1.388（0.908，2.121） | 9 |  |
|  |  |  |  |  |  |  |  |  |  |  |  |  |  |  |
