# Supplementary material for: Right atrial volume index and right atrial volume predict atrial fibrillation recurrence: A meta-analysis
Source: PLoS One. 2024 Dec 16;19(12):e0315590. doi: 10.1371/journal.pone.0315590 (PMC11649108; doi:10.1371/journal.pone.0315590)
Supplement: S3 Table — (DOCX) [file pone.0315590.s003.docx]

| **S3 Table.** Characteristics of included studies （RVA） | | | | | | | | | | |  |
| --- | --- | --- | --- | --- | --- | --- | --- | --- | --- | --- | --- |
| Study | Year | Country | Disease status | Sample | Age (y) | Male，% | Hypertension，% | Diabetes，% | Dyslipidemia，% | Heart Failure，% |  |
|  |  |  |  |  |  |  |  |  |  |  |  |
| Pan T ^[21]^ | 2023 | China | PaAF and PeAF | 297 | 61.1±11 | 60.2 | 55.6 | 17.1 | 22.9 | 23.9 |  |
|  |  |  |  |  |  |  |  |  |  |  |  |
| Tomaselli M ^[26]^ | 2023 | Italy | PeAF | 132 | 72±10 | 55 | 81 | 11 | N/A | N/A |  |
|  |  |  |  |  |  |  |  |  |  |  |  |
| Gunturiz-Beltrán C ^[27]^ | 2022 | Spain | PaAF and PeAF | 100 | 59±11 | 70 | 54 | 9 | N/A | N/A |  |
|  |  |  |  |  |  |  |  |  |  |  |  |
| Takagi T ^[28]^ | 2021 | Japan | Drug-refractory AF | 213 | 64.7±10.5 | 78 | 49 | 18 | N/A | 15 |  |
|  |  |  |  |  |  |  |  |  |  |  |  |
| Kumagai Y ^[29]^ | 2018 | Japan | PaAF | 100 | 59.8 ± 9.5 | 74 | 57 | 9 | N/A | 4 |  |
|  |  |  |  |  |  |  |  |  |  |  |  |
| Zhao L ^[30]^ | 2013 | China | PeAF | 208 | 63±8 | 66 | 55 | 15 | N/A | N/A |  |
|  |  |  |  |  |  |  |  |  |  |  |  |
| Akutsu Y ^[31]^ | 2011 | Japan | PaAF | 65 | 59±10 | 81.5 | 47.7 | 14.8 | 29.2 | N/A |  |
|  |  |  |  |  |  |  |  |  |  |  |  |
