# Supplementary material for: Right atrial volume index and right atrial volume predict atrial fibrillation recurrence: A meta-analysis
Source: PLoS One. 2024 Dec 16;19(12):e0315590. doi: 10.1371/journal.pone.0315590 (PMC11649108; doi:10.1371/journal.pone.0315590)
Supplement: S4 Table — (DOCX) [file pone.0315590.s004.docx]

| **S4 Table.** Characteristics of included studies （RVA） | | | | | | | | | | | | | |  |
| --- | --- | --- | --- | --- | --- | --- | --- | --- | --- | --- | --- | --- | --- | --- |
| Study | Year | Disease status | Surgical method | Imaging used | Mean follow-up months | Recurrence detection method | Number of people with recurrence of atrial fibrillation，n | Number of people without recurrence of atrial fibrillation，n | Mean RAV，ml | Mean RAV，ml | | HR/OR | NOS |  |
|  |  |  |  |  |  |  |  |  |  | Recurrence | No Recurrence |  |  |  |
| Pan T ^[21]^ | 2023 | PaAF and PeAF | RFA | CT | 12 | Holter and ECG | 83 | 214 | 80.96 ± 24.8 | 92.19±33.94 | 76.46±20.22 | 1.011（0.991，1.031） | 8 |  |
|  |  |  |  |  |  |  |  |  |  |  |  |  |  |  |
| Tomaselli M ^[26]^ | 2023 | PeAF | ECV | echocardiography | 12 | Holter and ECG | 63 | 69 | 35±14 | 37±15 | 33±13 | 1.01（0.99，1.03） | 6 |  |
|  |  |  |  |  |  |  |  |  |  |  |  |  |  |  |
| Gunturiz-Beltrán C ^[27]^ | 2022 | PaAF and PeAF | PVI | CMR | 23.6 | Holter and ECG | 50 | 50 | 126.8 ± 31.6 | N/A | N/A | 1.07（1.00，1.14） | 7 |  |
|  |  |  |  |  |  |  |  |  |  |  |  |  |  |  |
| Takagi T ^[28]^ | 2021 | Drug-refractory AF | PVI | CT | 13±3.8 | Holter and ECG | 41 | 172 | 111 ± 43.7 | 145±62.3 | 103±32.9 | 1.011（1.002，1.021） | 9 |  |
|  |  |  |  |  |  |  |  |  |  |  |  |  |  |  |
| Kumagai Y ^[29]^ | 2018 | PaAF | PVI | CMR | 8 | Holter and ECG | 23 | 77 | 88.46 ± 21.48 | 109.4 ± 27.0 | 82.2 ± 19.6 | 3.70 (2.07-7.43) | 9 |  |
|  |  |  |  |  |  |  |  |  |  |  |  |  |  |  |
| Zhao L ^[30]^ | 2013 | PeAF | PVI | echocardiography | 12 | Holter | 34 | 174 | N/A | N/A | N/A | 2.80（1.11，7.07） | 8 |  |
|  |  |  |  |  |  |  |  |  |  |  |  |  |  |  |
| Akutsu Y ^[31]^ | 2011 | PaAF | PV-CA | MDCT | 6 | Holter | 16 | 49 | 83.3±30.3 | 118±41 | 72±26 | 1.04（1.02，1.07） | 9 |  |
|  |  |  |  |  |  |  |  |  |  |  |  |  |  |  |
