# Supplementary material for: Right atrial volume index and right atrial volume predict atrial fibrillation recurrence: A meta-analysis
Source: PLoS One. 2024 Dec 16;19(12):e0315590. doi: 10.1371/journal.pone.0315590 (PMC11649108; doi:10.1371/journal.pone.0315590)
Supplement: S5 Table — (DOCX) [file pone.0315590.s005.docx]

**S5 Table.** Subgroup analysis of the relationship between the risk of atrial fibrillation recurrence and RAVI levels in patients with atrial fibrillation who underwent electrical cardioversion or radiofrequency ablation

| **Subgroups** |  | **Overall effect** | | |  | **Heterogeneity** | |
| --- | --- | --- | --- | --- | --- | --- | --- |
|  |  | **Studies，n** | **OR (95% CI)** | **P value** |  | **I^2^ (%)** | **P** |
| **All** |  | 11 | 1.06（1.02，1.11） | 0.009 |  | 69.3 | ＜0.001 |
|  |  |  |  |  |  |  |  |
| **Study location** |  |  |  |  |  |  |  |
| Asia |  | 8 | 1.16（1.03，1.29） | 0.011 |  | 74.6 | ＜0.001 |
| Europe |  | 2 | 1.02（1.00，1.04） | 0.113 |  | 0 | 0.727 |
| North America |  | 1 | 1.56（0.68，3.58） | 0.295 |  | - | - |
| **Year of publication** |  |  |  |  |  |  |  |
| ≥2014 |  | 7 | 1.06（1.01，1.10） | 0.008 |  | 67.6 | 0.005 |
| ＜2014 |  | 4 | 1.18（0.89，1.56） | 0.255 |  | 78.4 | 0.003 |
| **Participants，n** |  |  |  |  |  |  |  |
| ≥100 |  | 5 | 1.19（1.05，1.34） | 0.005 |  | 74.1 | 0.004 |
| ＜100 |  | 6 | 1.01（0.95，1.08） | 0.766 |  | 58.4 | 0.035 |
| **Age，year** |  |  |  |  |  |  |  |
| ≥60 |  | 2 | 1.04（1.02，1.06） | ＜0.001 |  | 0 | 0.342 |
| ＜60 |  | 9 | 1.10（1.02，1.18） | 0.013 |  | 73.9 | ＜0.001 |
| **Male，%** |  |  |  |  |  |  |  |
| ≥80 |  | 4 | 1.01（0.95，1.07） | 0.771 |  | 63 | 0.044 |
| ＜80 |  | 7 | 1.23（1.08，1.41） | 0.002 |  | 68.9 | 0.004 |
| **Mean follow-up，months** | |  |  |  |  |  |  |
| ≥12 |  | 5 | 1.09（0.96，1.23） | 0.19 |  | 74 | 0.004 |
| ＜12 |  | 6 | 1.09（1.01，1.17） | 0.034 |  | 68.3 | 0.007 |
| **Mean RAVI，ml/m^2^** |  |  |  |  |  |  |  |
| ≥82 |  | 5 | 1.16（0.98，1.37） | 0.09 |  | 79.1 | ＜0.001 |
| ＜82 |  | 6 | 1.09（0.96，1.23） | 0.034 |  | 44.7 | 0.108 |
| **Hypertension，%** |  |  |  |  |  |  |  |
| ≥45 |  | 6 | 1.03（1.00，1.06） | 0.034 |  | 44.7 | 0.108 |
| ＜45 |  | 5 | 1.16（0.98，1.37） | 0.09 |  | 79.1 | ＜0.001 |
| **Diabetes，%** |  |  |  |  |  |  |  |
| ≥5.5 |  | 4 | 1.03（0.90，1.18） | 0.658 |  | 69.2 | 0.021 |
| ＜5.5 |  | 5 | 1.28（1.17，1.42） | ＜0.001 |  | 0 | 0.845 |
| N/A |  | 2 | 1.02（1.00，1.04） | 0.113 |  | 0 | 0.727 |
| **Dyslipidemia，%** |  |  |  |  |  |  |  |
| ≥12 |  | 5 | 1.19（1.04，1.37） | 0.011 |  | 72 | 0.006 |
| ＜12 |  | 4 | 1.18（0.89，1.56） | 0.255 |  | 78.4 | 0.003 |
| N/A |  | 2 | 1.02（1.00，1.04） | 0.113 |  | 0 | 0.727 |
| **Heart Failure，%** |  |  |  |  |  |  |  |
| ≥4 |  | 5 | 1.19（1.04，1.37） | 0.011 |  | 72 | 0.006 |
| ＜4 |  | 4 | 1.18（0.89，1.56） | 0.255 |  | 78.4 | 0.003 |
| N/A |  | 2 | 1.02（1.00，1.04） | 0.113 |  | 0 | 0.727 |
