# Supplementary material for: Right atrial volume index and right atrial volume predict atrial fibrillation recurrence: A meta-analysis
Source: PLoS One. 2024 Dec 16;19(12):e0315590. doi: 10.1371/journal.pone.0315590 (PMC11649108; doi:10.1371/journal.pone.0315590)
Supplement: S6 Table — (DOCX) [file pone.0315590.s006.docx]

**S6 Table.** Subgroup analysis of the relationship between the risk of atrial fibrillation recurrence and RAV levels in patients with atrial fibrillation who underwent electrical cardioversion or radiofrequency ablation

| **Subgroups** |  | **Overall effect** | | |  | **Heterogeneity** | |
| --- | --- | --- | --- | --- | --- | --- | --- |
|  |  | **Studies，n** | **OR (95% CI)** | **P value** |  | **I^2^ ，%** | **P** |
| **All** |  | 7 | 1.02（1.00，1.05） | 0.047 |  | 78.3 | ＜0.001 |
|  |  |  |  |  |  |  |  |
| **Study location** |  |  |  |  |  |  |  |
| Asia |  | 5 | 1.03（0.99，1.06） | 0.152 |  | 84.1 | ＜0.001 |
| Europe |  | 2 | 1.03（0.98，1.08） | 0.273 |  | 60.5 | 0.111 |
| **Year of publication** | |  |  |  |  |  |  |
| ≥2022 |  | 3 | 1.01（1.00，1.03） | 0.102 |  | 23.1 | 0.272 |
| ＜2022 |  | 4 | 1.04（0.98，1.11） | 0.194 |  | 88 | ＜0.001 |
| **Participants，n** |  |  |  |  |  |  |  |
| ≥200 |  | 3 | 1.01（0.99，1.03） | 0.274 |  | 57 | 0.098 |
| ＜200 |  | 4 | 1.04（0.99，1.10） | 0.112 |  | 85.4 | ＜0.001 |
| **Age，year** |  |  |  |  |  |  |  |
| ≥63 |  | 3 | 1.01（0.99，1.03） | 0.295 |  | 57.1 | 0.097 |
| ＜63 |  | 4 | 1.04（0.99，1.10） | 0.107 |  | 85.2 | ＜0.001 |
| **Male，%** |  |  |  |  |  |  |  |
| ≥70 |  | 4 | 1.04（0.99，1.09） | 0.093 |  | 86.7 | ＜0.001 |
| ＜70 |  | 3 | 1.01（0.98，1.04） | 0.406 |  | 57.1 | 0.097 |
| **Mean follow-up，months** | |  |  |  |  |  |  |
| ≥13 |  | 2 | 1.03（0.98，1.08） | 0.264 |  | 61.8 | 0.106 |
| ＜13 |  | 5 | 1.03（0.99，1.07） | 0.194 |  | 83.6 | ＜0.001 |
| **Mean RAV，ml** |  |  |  |  |  |  |  |
| ≥100 |  | 2 | 1.03（0.98，1.08） | 0.264 |  | 61.8 | 0.106 |
| ＜100 |  | 4 | 1.02（0.99，1.06） | 0.204 |  | 84.8 | ＜0.001 |
| N/A |  | 1 | 2.8（1.11，7.07） | 0.029 |  | - | - |
| **Hypertension，%** |  |  |  |  |  |  |  |
| ≥55 |  | 4 | 1.03（0.97，1.09） | 0.396 |  | 85.4 | ＜0.001 |
| ＜55 |  | 3 | 1.03（1.00，1.06） | 0.04 |  | 71.1 | 0.031 |
| **Diabetes，%** |  |  |  |  |  |  |  |
| ≥15 |  | 3 | 1.01（0.99，1.03） | 0.274 |  | 57 | 0.098 |
| ＜15 |  | 4 | 1.04（0.99，1.10） | 0.112 |  | 85.4 | ＜0.001 |
