# Supplementary material for: Right atrial volume index and right atrial volume predict atrial fibrillation recurrence: A meta-analysis
Source: PLoS One. 2024 Dec 16;19(12):e0315590. doi: 10.1371/journal.pone.0315590 (PMC11649108; doi:10.1371/journal.pone.0315590)
Supplement: S8 Table — (DOCX) [file pone.0315590.s008.docx]

**S8 Table.** A numbered table of all studies identified in the literature search.

1. 谢倩, 韩嘉明, and 马玉兰, *右心房在术后心房颤动复发中的研究进展 %J 中国心血管病研究 %J Chinese Journal of Cardiovascular Research.* 2024. **22**(8): p. 684-689. （The reason for exclusion: review）

2. 谢倩, 韩嘉明, and 马玉兰, *右心房在术后心房颤动复发中的研究进展 %J 中国心血管病研究.* 2024. **22**(08): p. 684-689. （The reason for exclusion: duplicate record）

3. 陈金兰. *右心房容积指数预测房颤合并高血压患者“一战式”手术后复发的临床价值*. in *2024第二届四川省体育科学大会论文报告会*. 2024. 中国四川成都. （The reason for exclusion: without relevant date）

4. Singh, S., et al., *AN UNFORTUNATE TWIST: SARCOMATOID PULMONARY SPINDLE CELL CARCINOMA*. 2024. p. A4352-A4353. （The reason for exclusion: meeting abstract）

5. Payami, B., et al., *Evaluation of the Effect of Conversion of Nonvalvular Atrial Fibrillation to Sinus Rhythm on Cardiac Remodeling.* Cureus, 2024. **16**(5): p. e60504. （The reason for exclusion: Irrelevant study outcome）

6. Payami, B., et al., *Evaluation of the Effect of Conversion of Nonvalvular Atrial Fibrillation to Sinus Rhythm on Cardiac Remodeling.* Cureus Journal of Medical Science, 2024. **16**(5): p. 15. （The reason for exclusion: duplicate record）

7. Pan, T., et al., *The role of the right atrial appendage and right atrium in post-radiofrequency ablation recurrence in different types of atrial fibrillation.* Clin Radiol, 2024. **79**(11): p. e1312-e1320. （The reason for exclusion: without relevant date）

8. Pan, T., et al., *The role of the right atrial appendage and right atrium in post-radiofrequency ablation recurrence in different types of atrial fibrillation.* Clinical Radiology, 2024. （The reason for exclusion: duplicate record）

9. Nodera, M., et al., *Epicardial adipose tissue density predicts the presence of atrial fibrillation and its recurrence after catheter ablation: three-dimensional reconstructed image analysis.* Heart Vessels, 2024. **39**(8): p. 696-705. （The reason for exclusion: Irrelevant study outcome）

10. Nodera, M., et al., *Epicardial adipose tissue density predicts the presence of atrial fibrillation and its recurrence after catheter ablation: three-dimensional reconstructed image analysis.* Heart and Vessels, 2024. **39**(8): p. 696-705. （The reason for exclusion: duplicate record）

11. Mouram, S., et al., *PO-03-156 ACUTE AND LONG-TERM LESION DURABILITY AND RECONNECTION PATTERNS FOLLOWING HYBRID ATRIAL FIBRILLATION ABLATION.* Heart Rhythm, 2024. **21**(5): p. S349. （The reason for exclusion: Irrelevant study outcome）

12. Mărgulescu, A.D., et al., *Combined Area of Left and Right Atria May Outperform Atrial Volumes as a Predictor of Recurrences after Ablation in Patients with Persistent Atrial Fibrillation-A Pilot Study.* Medicina (Kaunas, Lithuania), 2024. **60**(1). (This document meets the inclusion criteria)

13. Liao, Y., et al., *Initial clinical experience of atrial fibrillation ablation guided by a cryoballoon-compatible, magnetic-based circular catheter.* J Cardiovasc Electrophysiol, 2024. **35**(1): p. 111-119. （The reason for exclusion: Irrelevant study outcome）

14. Lehel, B., et al., *A High Coronary Calcium Score And Increased Epicardial Fat Assessed By Ccta Are Associated With Atrial Fibrillation Recurrence Following Catheter Ablation.* Journal of Cardiovascular Computed Tomography, 2024. **18**(1): p. S19. （The reason for exclusion: Irrelevant study outcome）

15. Kuo, L., et al., *Deep learning-based workflow for automatic extraction of atria and epicardial adipose tissue on cardiac computed tomography in atrial fibrillation.* Journal of the Chinese Medical Association, 2024. **87**(5): p. 471-479. （The reason for exclusion: Irrelevant study outcome）

16. Kuo, L., et al., *Feasibility of Auto-Quantified Epicardial Adipose Tissue in Predicting Atrial Fibrillation Recurrence After Catheter Ablation.* Circ J, 2024. **88**(7): p. 1089-1098. （The reason for exclusion: Irrelevant study outcome）

17. Kim, M., et al., *The Effects of Radiofrequency Catheter Ablation for Atrial Fibrillation on Right Ventricular Function.* Korean Circulation Journal, 2024. **54**(4): p. 203-217. （The reason for exclusion: Irrelevant study outcome）

18. Geršak, B., et al., *Long-Term Outcomes after Convergent Procedure for Atrial Fibrillation.* Journal of Clinical Medicine, 2024. **13**(18). （The reason for exclusion: Irrelevant study outcome）

19. Deveaux, H., et al., *REFRACTORY ORTHODROMIC SUPRAVENTRICULAR TACHYCARDIA WITH WOLFF-PARKINSON-WHITE SYNDROME*. 2024. p. A368-A369. （The reason for exclusion: Irrelevant study outcome）

20. Chandrasekhar, S., et al., *PO-02-034 CARDIOGENIC SHOCK AND PNEUMOPERICARDIUM: A RARE PRESENTATION OF ATRIOESOPHAGEAL FISTULA.* Heart Rhythm, 2024. **21**(5): p. S320. （The reason for exclusion: Irrelevant study outcome）

21. Androsov, A. and T. Leo, *ABLATION OF PERSISTENT ATRIAL FIBRILLATION IN A PATIENT WITH CARDIOGENIC SHOCK ON VA ECMO.* Journal of the American College of Cardiology, 2024. **83**(13): p. 3968. （The reason for exclusion: Irrelevant study outcome）

22. Alderete, J., et al., *The Ablate-by-LAWT multicentre prospective study: Personalized paroxysmal atrial fibrillation ablation with ablation index adapted to local left atrial wall thickness.* Journal of Interventional Cardiac Electrophysiology, 2024: p. 11. （The reason for exclusion: Irrelevant study outcome）

23. Alderete, J., et al., *The Ablate-by-LAWT multicentre prospective study: Personalized paroxysmal atrial fibrillation ablation with ablation index adapted to local left atrial wall thickness.* Journal of Interventional Cardiac Electrophysiology, 2024. （The reason for exclusion: Irrelevant study outcome）

24. Abadir, S., et al., *POSTCARDIOVERSION FOR ATRIAL FLUTTER COMPLICATED WITH SINUS BRADYCARDIA AND PAUSES: A CASE OF SICK SINUS SYNDROME REQUIRING PACEMAKER IMPLANTATION*. 2024. p. A799-A800. （The reason for exclusion: case report）

25. 周世强, 史喜德, and 李飞, *心房功能性三尖瓣反流研究进展 %J 心脏杂志.* 2023. **35**(03): p. 355-358. （The reason for exclusion: review）

26. 赵耀, *冷冻顶部线联合肺静脉隔离治疗持续性心房颤动的前瞻性随机对照研究*. 2023. （The reason for exclusion: Irrelevant study outcome）

27. 张付涛, *完全性右束支传导阻滞与心房颤动的相关性及其对房颤导管消融术后复发的预测价值*. 2023. （The reason for exclusion: Irrelevant study outcome）

28. 杨梦琪, et al., *超声评估心房颤动伴房室瓣反流的研究进展 %J 中国医学影像学杂志.* 2023. **31**(04): p. 429-432. （The reason for exclusion: review）

29. 杨梦琪, *房颤患者二尖瓣与左心重构相关性探究*. 2023. （The reason for exclusion: Irrelevant study outcome）

30. 徐鑫, *右心房、右心室应变在持续性房颤患者射频消融术中的应用价值*. 2023. （The reason for exclusion: without relevant date）

31. 吴碧君, 赵萍, and 杨瑶琳, *经胸实时三维超声心动图评估心房颤动合并功能性三尖瓣反流患者右心形态结构改变的研究 %J 中国循环杂志.* 2023. **38**(11): p. 1127-1133. （The reason for exclusion: Irrelevant study outcome）

32. 王艳晶, *基于CMR的心房功能多元化分析及其在心房重构评估中的应用研究*. 2023. （The reason for exclusion: Irrelevant study outcome）

33. 史发超, *功能性三尖瓣反流与房颤消融术后复发的相关性研究*. 2023. （The reason for exclusion: Irrelevant study outcome）

34. 马航宇, et al., *超声心动图评价心房颤动患者心房结构和功能研究进展 %J 中华实用诊断与治疗杂志.* 2023. **37**(10): p. 1077-1080. （The reason for exclusion: review）

35. 罗方远, et al., *通过腔内心电图测量心房传导时间对心房颤动复发的预测价值 %J 中国循证心血管医学杂志 %J Chinese Journal of Evidence-Bases Cardiovascular Medicine.* 2023. **15**(8): p. 932-935,945. （The reason for exclusion: Irrelevant study outcome）

36. 林儒峥, 韩坤元, and 朱材忠, *社区初诊心房颤动病人早期自发转为窦性心律的影响因素分析 %J 中西医结合心脑血管病杂志.* 2023. **21**(04): p. 723-727. （The reason for exclusion: Irrelevant study outcome）

37. 李晓磊, *急诊经皮冠状动脉介入术后患者新发房颤的危险因素及预后分析*. 2023. （The reason for exclusion: Irrelevant study outcome）

38. 贾晓艳, *基于心脏结构和功能—射血分数保留心力衰竭合并心房颤动的表型分析*. 2023. （The reason for exclusion: Irrelevant study outcome）

39. 胡秋明, et al., *23例微创双房消融路径治疗孤立性心房颤动的临床经验 %J 心肺血管病杂志.* 2023. **42**(03): p. 252-256. （The reason for exclusion: Irrelevant study outcome）

40. 补锐铃, *消融术前PLR、NLR、NT-pro BNP对于房颤射频消融术后复发的预测价值*. 2023. （The reason for exclusion: Irrelevant study outcome）

41. Tomaselli, M., et al., *Incremental Value of Right Atrial Strain Analysis to Predict Atrial Fibrillation Recurrence After Electrical Cardioversion.* J Am Soc Echocardiogr, 2023. **36**(9): p. 945-955. (This document meets the inclusion criteria)

42. Tomaselli, M., et al., *Incremental Value of Right Atrial Strain Analysis to Predict Atrial Fibrillation Recurrence After Electrical Cardioversion.* Journal of the American Society of Echocardiography, 2023. **36**(9): p. 945-955. （The reason for exclusion: duplicate record）

43. Shigeta, T., et al., *How to perform effective cryoballooon ablation of the left atrial roof: Considerations after experiencing more than 1000 cases.* J Cardiovasc Electrophysiol, 2023. **34**(12): p. 2484-2492. （The reason for exclusion: Irrelevant study outcome）

44. Segan, L., et al., *Posterior Wall Isolation Improves Outcomes for Persistent AF With Rapid Posterior Wall Activity.* Jacc-Clinical Electrophysiology, 2023. **9**(12): p. 2536-2546. （The reason for exclusion: Irrelevant study outcome）

45. Segan, L., et al., *Posterior Wall Isolation Improves Outcomes for Persistent AF With Rapid Posterior Wall Activity: CAPLA Substudy.* JACC Clin Electrophysiol, 2023. **9**(12): p. 2536-2546. （The reason for exclusion: duplicate record）

46. Sakata, K., et al., *The spatiotemporal electrogram dispersion ablation targeting rotors is more effective for elderly patients than non-elderly population.* Journal of Arrhythmia, 2023. **39**(3): p. 315-326. （The reason for exclusion: Irrelevant study outcome）

47. Qi, D. and J.J. Zhang, *Relationship between anatomical characteristics of pulmonary veins and atrial fibrillation recurrence after radiofrequency catheter ablation: a systematic review and meta-analysis.* Frontiers in Cardiovascular Medicine, 2023. **10**: p. 10. （The reason for exclusion: Irrelevant study outcome）

48. Pongratz, J., et al., *Left atrial appendage volume is an independent predictor of atrial arrhythmia recurrence following cryoballoon pulmonary vein isolation in persistent atrial fibrillation.* Frontiers in Cardiovascular Medicine, 2023. **10**. （The reason for exclusion: Irrelevant study outcome）

49. Pan, T., et al., *Association of quantitative computed tomography-based right atrial appendage and right atrium parameters with postradiofrequency ablation recurrence of atrial fibrillation.* Quant Imaging Med Surg, 2023. **13**(6): p. 3802-3815. (This document meets the inclusion criteria)

50. Pan, T., et al., *Association of quantitative computed tomography-based right atrial appendage and right atrium parameters with postradiofrequency ablation recurrence of atrial fibrillation.* Quantitative Imaging in Medicine and Surgery, 2023. **13**(6): p. 3802-3815. （The reason for exclusion: duplicate record）

51. Pan, T., et al., *Association of quantitative computed tomography-based right atrial appendage and right atrium parameters with postradiofrequency ablation recurrence of atrial fibrillation.* Quantitative Imaging in Medicine and Surgery, 2023. **13**(6). （The reason for exclusion: duplicate record）

52. Osorio, J., et al., *HIGH-FREQUENCY LOW-TIDAL VOLUME MECHANICAL VENTILATION IS ASSOCIATED WITH IMPROVED ACUTE AND LONG-TERM OUTCOMES IN PATIENTS UNDERGOING CATHETER ABLATION FOR ATRIAL FIBRILLATION ABLATION.* Heart Rhythm, 2023. **20**(5): p. S219-S220. （The reason for exclusion: Irrelevant study outcome）

53. Kronenberger, R., et al., *Stiff left atrial syndrome with pulmonary veins occlusion after percutaneous radiofrequency ablation: a life-long complication that can lead to heart transplantation.* J Cardiothorac Surg, 2023. **18**(1): p. 181. （The reason for exclusion: Irrelevant study outcome）

54. Kronenberger, R., et al., *Stiff left atrial syndrome with pulmonary veins occlusion after percutaneous radiofrequency ablation: a life-long complication that can lead to heart transplantation.* Journal of cardiothoracic surgery, 2023. **18**(1): p. 181. （The reason for exclusion: duplicate record）

55. Kronenberger, R., et al., *Stiff left atrial syndrome with pulmonary veins occlusion after percutaneous radiofrequency ablation: a life-long complication that can lead to heart transplantation.* Journal of Cardiothoracic Surgery, 2023. **18**(1): p. 5. （The reason for exclusion: duplicate record）

56. Hopman, L.H.G.A., et al., *Right atrial function and fibrosis in relation to successful atrial fibrillation ablation.* European Heart Journal Cardiovascular Imaging, 2023. **24**(3): p. 336-345. （The reason for exclusion: Irrelevant study outcome）

57. Hopman, L., et al., *Right atrial function and fibrosis in relation to successful atrial fibrillation ablation.* Eur Heart J Cardiovasc Imaging, 2023. **24**(3): p. 336-345. （The reason for exclusion: duplicate record）

58. Falasconi, G., et al., *Personalized pulmonary vein antrum isolation guided by left atrial wall thickness for persistent atrial fibrillation.* Europace, 2023. **25**(5): p. 15. （The reason for exclusion: Irrelevant study outcome）

59. Falasconi, G., et al., *Personalized pulmonary vein antrum isolation guided by left atrial wall thickness for persistent atrial fibrillation.* Europace, 2023. **25**(5). （The reason for exclusion: duplicate record）

60. Doring, C., et al., *The Impact of Right Atrial Size to Predict Success of Direct Current Cardioversion in Patients With Persistent Atrial Fibrillation.* Korean Circulation Journal, 2023. **53**(5): p. 331-343. （The reason for exclusion: Irrelevant study outcome）

61. Bordi, L.L., et al., *Association of Atrial Fibrillation Recurrence with Right Coronary Atherosclerosis and Increased Left Arterial Epicardial Fat Following Catheter Ablation-Results of a Multimodality Study.* Life-Basel, 2023. **13**(9): p. 18. （The reason for exclusion: Irrelevant study outcome）

62. 张付涛, et al., *完全性右束支传导阻滞与右心房增大的相关性研究 %J 中国心血管病研究.* 2022. **20**(08): p. 711-714. （The reason for exclusion: Irrelevant study outcome）

63. 袁含茵, et al., *双心房应变预测阵发性心房颤动导管消融术后复发 %J 中国介入心脏病学杂志.* 2022. **30**(03): p. 173-179. （The reason for exclusion: without relevant date）

64. 孙广龙, et al., *完整迷宫Ⅳ手术治疗心脏疾病合并心房颤动术后近中期疗效分析 %J 心肺血管病杂志 %J Journal of Cardiovascular and Pulmonary Diseases.* 2022. **41**(12): p. 1258-1262. （The reason for exclusion: Irrelevant study outcome）

65. 宋文华, et al., *老年心房颤动患者冷冻球囊消融术后复发相关影响因素分析 %J 中华老年心脑血管病杂志.* 2022. **24**(12): p. 1279-1282. （The reason for exclusion: Irrelevant study outcome）

66. 宋倩 and 刘永铭, *射血分数保留性心力衰竭合并心房颤动的右心室及右心房结构及功能 %J 心血管病学进展.* 2022. **43**(08): p. 722-725. （The reason for exclusion: review）

67. 马雅菁, *左、右房应变及三维容积指数对阵发性房颤射频消融后房颤复发的预测*. 2022. （The reason for exclusion: without relevant date）

68. 刘丹妮, 冉海涛, and 敖梦, *实时三维超声心动图和斑点追踪成像评价房颤患者心房结构和功能的研究进展 %J 中国医学影像学杂志.* 2022. **30**(05): p. 518-523. （The reason for exclusion: review）

69. 方浩, *预测心房颤动患者导管消融术后复发的列线图模型*. 2022. （The reason for exclusion: Irrelevant study outcome）

70. Zheng, Z., et al., *Box lesion or bi-atrial lesion set for atrial fibrillation during thoracoscopic epicardial ablation.* Interactive Cardiovascular and Thoracic Surgery, 2022. **34**(1): p. 1-8. （The reason for exclusion: Irrelevant study outcome）

71. Yang, L., et al., *Sacubitril/valsartan attenuates atrial structural remodelling in atrial fibrillation patients.* Esc Heart Failure, 2022. **9**(4): p. 2428-2434. （The reason for exclusion: Irrelevant study outcome）

72. Yang, L., et al., *Sacubitril/valsartan attenuates atrial structural remodelling in atrial fibrillation patients.* ESC Heart Fail, 2022. **9**(4): p. 2428-2434. （The reason for exclusion: duplicate record）

73. Yang, L., et al., *Sacubitril/valsartan attenuates atrial structural remodelling in atrial fibrillation patients.* ESC heart failure, 2022. **9**(4): p. 2428‐2434. （The reason for exclusion: duplicate record）

74. Yang, L., et al., *Sacubitril/valsartan attenuates atrial structural remodelling in atrial fibrillation patients.* ESC Heart Failure, 2022. **9**(4): p. 2428-2434. （The reason for exclusion: duplicate record）

75. Shono, A., et al., *Ability of Left Atrial Distensibility After Radiofrequency Catheter Ablation to Predict Recurrence of Atrial Fibrillation.* American Journal of Cardiology, 2022. **181**: p. 59-65. （The reason for exclusion: Irrelevant study outcome）

76. Rajendra, A., et al., *PO-667-03 RADIOFREQUENCY ABLATION OF PAROXYSMAL ATRIAL FIBRILLATION IN OCTOGENARIANS: INSIGHTS FROM A MULTICENTER REGISTRY (REAL-AF).* Heart Rhythm, 2022. **19**(5): p. S308. （The reason for exclusion: Irrelevant study outcome）

77. Premont, A., et al., *Fundamentals of arrhythmogenic mechanisms and treatment strategies for equine atrial fibrillation.* Equine Veterinary Journal, 2022. **54**(2): p. 262-282. （The reason for exclusion: animal experiment）

78. Liebregts, M., et al., *Initial experience with AcQMap catheter for treatment of persistent atrial fibrillation and atypical atrial flutter.* Netherlands Heart Journal, 2022. **30**(5): p. 273-281. （The reason for exclusion: Irrelevant study outcome）

79. Li, Y., et al., *Value of echocardiography in evaluating efficacy of radiofrequency catheter ablation in patients with atrial fibrillation.* American Journal of Translational Research, 2022. **14**(3): p. 1778-1787. （The reason for exclusion: Irrelevant study outcome）

80. Li, Y., et al., *Value of echocardiography in evaluating efficacy of radiofrequency catheter ablation in patients with atrial fibrillation.* Am J Transl Res, 2022. **14**(3): p. 1778-1787. （The reason for exclusion: duplicate record）

81. Li, Y., et al., *Value of echocardiography in evaluating efficacy of radiofrequency catheter ablation in patients with atrial fibrillation.* American Journal of Translational Research, 2022. **14**(3): p. 1778-1787. （The reason for exclusion: duplicate record）

82. Labarbera, M.A., et al., *New Radiomic Markers of Pulmonary Vein Morphology Associated With Post-Ablation Recurrence of Atrial Fibrillation.* Ieee Journal of Translational Engineering in Health and Medicine, 2022. **10**: p. 9. （The reason for exclusion: Irrelevant study outcome）

83. Labarbera, M.A., et al., *New Radiomic Markers of Pulmonary Vein Morphology Associated With Post-Ablation Recurrence of Atrial Fibrillation.* IEEE J Transl Eng Health Med, 2022. **10**: p. 1800209. （The reason for exclusion: duplicate record）

84. Labarbera, M.A., et al., *New Radiomic Markers of Pulmonary Vein Morphology Associated with Post-Ablation Recurrence of Atrial Fibrillation.* IEEE Journal of Translational Engineering in Health and Medicine, 2022. **10**. （The reason for exclusion: duplicate record）

85. Kumagai, K., et al., *Predictors of recurrence of atrial tachyarrhythmias after pulmonary vein isolation by functional and structural mapping of nonparoxysmal atrial fibrillation.* Journal of Arrhythmia, 2022. **38**(1): p. 86-96. （The reason for exclusion: Irrelevant study outcome）

86. Knecht, S., et al., *Efficacy and safety of a novel cryoballoon ablation system: multicentre comparison of 1-year outcome.* Europace, 2022. **24**(12): p. 1926-1932. （The reason for exclusion: Irrelevant study outcome）

87. Knecht, S., et al., *Efficacy and safety of a novel cryoballoon ablation system: multicentre comparison of 1-year outcome.* Europace, 2022. **24**(12): p. 1926-1932. （The reason for exclusion: duplicate record）

88. Kiliszek, M., et al., *Left atrial function parameters as predictors of atrial fibrillation recurrence after pulmonary vein isolation.* European Heart Journal, 2022. **43**: p. 606. （The reason for exclusion: Irrelevant study outcome）

89. Khalyfa, A., et al., *THROMBUS BEYOND US: A RARE MANIFESTATION OF LUNG ADENOCARCINOMA IN THE FORM OF TUMOR MICROTHROMBI.* Chest, 2022. **162**(4): p. A107-A108. （The reason for exclusion: case report）

90. Kalinsek, T.P. and D. Zizek, *Right-sided approach to left bundle branch area pacing combined with atrioventricular node ablation in a patient with persistent left superior vena cava and left bundle branch block: a case report.* Bmc Cardiovascular Disorders, 2022. **22**(1): p. 5. （The reason for exclusion: Irrelevant study outcome）

91. Jiang, W., et al., *Role and mechanism of lncRNA under magnetic nanoparticles in atrial autonomic nerve remodeling during radiofrequency ablation of recurrent atrial fibrillation.* Bioengineered, 2022. **13**(2): p. 4173-4184. （The reason for exclusion: Irrelevant study outcome）

92. Gunturiz-Beltrán, C., et al., *Progressive and Simultaneous Right and Left Atrial Remodeling Uncovered by a Comprehensive Magnetic Resonance Assessment in Atrial Fibrillation.* J Am Heart Assoc, 2022. **11**(20): p. e026028. (This document meets the inclusion criteria)

93. Ding, L., et al., *Angiographic Characteristics of the Vein of Marshall in Patients with and without Atrial Fibrillation.* Journal of Clinical Medicine, 2022. **11**(18): p. 11. （The reason for exclusion: Irrelevant study outcome）

94. Celik, E., et al., *Is there an association between left atrial outpouching structures and recurrence of atrial fibrillation after catheter ablation?* PLoS One, 2022. **17**(10): p. e0276369. （The reason for exclusion: Irrelevant study outcome）

95. Celik, E., et al., *Is there an association between left atrial outpouching structures and recurrence of atrial fibrillation after catheter ablation?* Plos One, 2022. **17**(10): p. 11. （The reason for exclusion: duplicate record）

96. Benjamin, M.M., et al., *Association of left atrial strain by cardiovascular magnetic resonance with recurrence of atrial fibrillation following catheter ablation.* J Cardiovasc Magn Reson, 2022. **24**(1): p. 3. （The reason for exclusion: Irrelevant study outcome）

97. Aksu, T., et al., *Procedural and short-term results of electroanatomic-mapping-guided ganglionated plexus ablation by first-time operators: A multicenter study.* J Cardiovasc Electrophysiol, 2022. **33**(1): p. 117-122. （The reason for exclusion: Irrelevant study outcome）

98. Aguilera, J., et al., *Outcomes of atrial fibrillation ablation in patients with or without silent pulmonary veins from prior ablation procedure.* J Cardiovasc Electrophysiol, 2022. **33**(9): p. 1994-2000. （The reason for exclusion: Irrelevant study outcome）

99. Aguilera, J., et al., *Outcomes of atrial fibrillation ablation in patients with or without silent pulmonary veins from prior ablation procedure.* Journal of Cardiovascular Electrophysiology, 2022. **33**(9): p. 1994-2000. （The reason for exclusion: duplicate record）

100. 邢长洋, et al., *欧洲心血管影像协会/美国超声心动图学会《二维斑点追踪超声心动图应用于左心房及右心应变成像的规范化共识》解读 %J 中华医学超声杂志(电子版).* 2021. **18**(12): p. 1135-1139. （The reason for exclusion: Irrelevant study outcome）

101. 孙金英 and 岳红梅, *阻塞性睡眠呼吸暂停与心房颤动的相关性研究进展 %J 中国医刊.* 2021. **56**(01): p. 31-33. （The reason for exclusion: review）

102. 马彦卓, et al., *以肺静脉电隔离为基础的个体化消融策略治疗持续性心房颤动的疗效研究 %J 实用心脑肺血管病杂志 %J Practical Journal of Cardiac Cerebral Pneumal and Vascular Disease.* 2021. **29**(10): p. 19-24. （The reason for exclusion: Irrelevant study outcome）

103. 罗大增, *青海地区非瓣膜性心房颤动患者危险因素分析*. 2021. （The reason for exclusion: Irrelevant study outcome）

104. 侯瀚林, *左心室肥大与持续性房颤射频消融术后复发的相关性研究*. 2021. （The reason for exclusion: Irrelevant study outcome）

105. Waldmann, V., et al., *Ablation par catheter dans les cardiopathies congenitales adultes: une perspective a 15 ans d'un centre expert.* Archives of Cardiovascular Diseases, 2021. **114**(6-7): p. 455-464. （The reason for exclusion: Irrelevant study outcome）

106. Urabayena, U.M., et al., *Anatomic characteristics of the left atrium in subjects undergoing radio frequency ablation for atrial fibrillation.* Radiologia, 2021. **63**(5): p. 391-399. （The reason for exclusion: Irrelevant study outcome）

107. Tsai, J.F., et al., *Acute Cardiac Tamponade as a Complication of Pulmonary Vein Isolation Ablation.* Cureus Journal of Medical Science, 2021. **13**(11): p. 6. （The reason for exclusion: Irrelevant study outcome）

108. Tsai, J., et al., *Acute Cardiac Tamponade as a Complication of Pulmonary Vein Isolation Ablation.* Cureus, 2021. **13**(11): p. e19572. （The reason for exclusion: duplicate record）

109. Teres, C., et al., *Personalized Atrial Fibrillation ablation by tailoring ablation index to the left atrial wall thickness. The “Ablate By-LAW” single center study.* Kardiovaskulare Medizin, 2021. **23**(SUPPL 29): p. 11S. （The reason for exclusion: Irrelevant study outcome）

110. Takagi, T., et al., *Impact of right atrial structural remodeling on recurrence after ablation for atrial fibrillation.* Journal of Arrhythmia, 2021. **37**(3): p. 597-606. (This document meets the inclusion criteria)

111. Seewöster, T., et al., *Biatrial volume ratio predicts low voltage areas in atrial fibrillation.* Clin Cardiol, 2021. **44**(11): p. 1560-1566. （The reason for exclusion: Irrelevant study outcome）

112. Ribeiro Da Silva, M., et al., *Redo ablation for atrial fibrillation recurrence post radiofrequency or cryoballoon ablation: A high volume single-centre experience.* Europace, 2021. **23**(SUPPL 3): p. iii111. （The reason for exclusion: Irrelevant study outcome）

113. Quinto, L., et al., *Cardiac magnetic resonance to predict recurrences after ventricular tachycardia ablation: septal involvement, transmural channels, and left ventricular mass.* Europace, 2021. **23**(9): p. 1437-1445. （The reason for exclusion: Irrelevant study outcome）

114. Nasso, G., et al., *The fate of patients after failed epicardial ablation of atrial fibrillation.* J Cardiothorac Surg, 2021. **16**(1): p. 249. （The reason for exclusion: Irrelevant study outcome）

115. Nasso, G., et al., *The fate of patients after failed epicardial ablation of atrial fibrillation.* Journal of cardiothoracic surgery, 2021. **16**(1): p. 249. （The reason for exclusion: duplicate record）

116. Nasso, G., et al., *Catheter, surgical, or hybrid procedure: what future for atrial fibrillation ablation?* J Cardiothorac Surg, 2021. **16**(1): p. 186. （The reason for exclusion: duplicate record）

117. Nasso, G., et al., *Catheter, surgical, or hybrid procedure: what future for atrial fibrillation ablation?* Journal of cardiothoracic surgery, 2021. **16**(1): p. 186. （The reason for exclusion: duplicate record）

118. Mirolo, A., et al., *Are routine cryoballoon procedural characteristics predictive of atrial arrhythmia recurrence in the long term?* Archives of Cardiovascular Diseases, 2021. **114**(2): p. 105-114. （The reason for exclusion: Irrelevant study outcome）

119. Martínez Urabayen, U., et al., *Anatomic characteristics of the left atrium in subjects undergoing radiofrequency ablation for atrial fibrillation.* Radiologia, 2021. **63**(5): p. 391-399. （The reason for exclusion: Irrelevant study outcome）

120. Martinez Urabayen, U., et al., *Anatomic characteristics of the left atrium in subjects undergoing radiofrequency ablation for atrial fibrillation.* Radiologia (Engl Ed), 2021. **63**(5): p. 391-399. （The reason for exclusion: duplicate record）

121. Martinez Urabayen, U., et al., *Anatomic characteristics of the left atrium in subjects undergoing radiofrequency ablation for atrial fibrillation.* Radiologia, 2021. **63**(5): p. 391-399. （The reason for exclusion: duplicate record）

122. Mangiafico, V., et al., *The role of CT in detecting AF substrate x2729.* Trends in Cardiovascular Medicine, 2021. **31**(8): p. 457-466. （The reason for exclusion: Irrelevant study outcome）

123. Maier, J., et al., *Cardiac Computed Tomography-Derived Left Atrial Volume Index as a Predictor of Long-Term Success of Cryo-Ablation in Patients With Atrial Fibrillation.* Am J Cardiol, 2021. **140**: p. 69-77. （The reason for exclusion: Irrelevant study outcome）

124. Maier, J., et al., *Cardiac Computed Tomography-Derived Left Atrial Volume Index as a Predictor of Long-Term Success of Cryo-Ablation in Patients With Atrial Fibrillation.* American Journal of Cardiology, 2021. **140**: p. 69-77. （The reason for exclusion: duplicate record）

125. Maenosono, R., et al., *Total atrial conduction time as a possible predictor of atrial fibrillation recurrence after catheter ablation for paroxysmal atrial fibrillation: relationship between electrical atrial remodeling and structural atrial remodeling time courses.* J Med Ultrason (2001), 2021. **48**(3): p. 295-306. （The reason for exclusion: Irrelevant study outcome）

126. Maenosono, R., et al., *Total atrial conduction time as a possible predictor of atrial fibrillation recurrence after catheter ablation for paroxysmal atrial fibrillation: relationship between electrical atrial remodeling and structural atrial remodeling time courses.* Journal of Medical Ultrasonics, 2021. **48**(3): p. 295-306. （The reason for exclusion: duplicate record）

127. Maenosono, R., et al., *Total atrial conduction time as a possible predictor of atrial fibrillation recurrence after catheter ablation for paroxysmal atrial fibrillation: relationship between electrical atrial remodeling and structural atrial remodeling time courses.* Journal of Medical Ultrasonics, 2021. **48**(3): p. 295-306. （The reason for exclusion: duplicate record）

128. Kim, S., et al., *The shape of the left lateral ridge as a predictor of long-term outcome of catheter ablation for atrial fibrillation based on clinical and experimental data.* International Journal of Cardiology, 2021. **329**: p. 91-98. （The reason for exclusion: Irrelevant study outcome）

129. Kawamura, I., et al., *How does the level of pulmonary venous isolation compare between pulsed field ablation and thermal energy ablation (radiofrequency, cryo, or laser)?* Europace, 2021. **23**(11): p. 1757-1766. （The reason for exclusion: Irrelevant study outcome）

130. Kawaji, T., et al., *Impact of catheter ablation for atrial fibrillation on cardiac disorders in patients with coexisting heart failure.* ESC Heart Fail, 2021. **8**(1): p. 670-679. （The reason for exclusion: Irrelevant study outcome）

131. Karantoumanis, I., et al., *Atrial conduction time associated predictors of recurrent atrial fibrillation.* Int J Cardiovasc Imaging, 2021. **37**(4): p. 1267-1277. （The reason for exclusion: Irrelevant study outcome）

132. Hermida, A., et al., *Results and Predictive Factors After One Cryoablation for Persistent Atrial Fibrillation.* American Journal of Cardiology, 2021. **159**: p. 65-71. （The reason for exclusion: Irrelevant study outcome）

133. Dikdan, S.J., et al., *Comparison of clinical and procedural outcomes between high-power short-duration, standard-power standard-duration, and temperature-controlled noncontact force guided ablation for atrial fibrillation.* J Cardiovasc Electrophysiol, 2021. **32**(3): p. 608-615. （The reason for exclusion: Irrelevant study outcome）

134. Brown, M.T., et al., *Outcomes of Manifest right free wall accessory pathway ablation: Data from a single center.* Journal of Atrial Fibrillation, 2021. **14**(1). （The reason for exclusion: Irrelevant study outcome）

135. Beyer, C., et al., *Structural Cardiac Remodeling in Atrial Fibrillation.* JACC Cardiovasc Imaging, 2021. **14**(11): p. 2199-2208. （The reason for exclusion: Irrelevant study outcome）

136. Beyer, C., et al., *Structural Cardiac Remodeling in Atrial Fibrillation.* JACC: Cardiovascular Imaging, 2021. **14**(11): p. 2199-2208. （The reason for exclusion: duplicate record）

137. 赵曙光, et al., *先天性心脏病合并房颤的改良右侧迷宫Ⅲ型手术疗效观察 %J 河北医药.* 2020. **42**(23): p. 3529-3533. （The reason for exclusion: Irrelevant study outcome）

138. 赵曙光, et al., *改良左侧迷宫Ⅲ型手术对不同病理生理分型风湿性二尖瓣病变房颤的效果 %J 河北医药.* 2020. **42**(24): p. 3733-3737. （The reason for exclusion: Irrelevant study outcome）

139. 岳凤捷, 金岩, and 王辉山, *心房功能性房室瓣反流的诊断特点 %J 中国心血管病研究.* 2020. **18**(12): p. 1130-1133. （The reason for exclusion: Irrelevant study outcome）

140. 孙广龙, et al., *Cox迷宫Ⅳ手术治疗心脏疾病合并心房颤动的近期疗效分析 %J 中国循环杂志 %J Chinese Circulation Journal.* 2020. **35**(1): p. 50-54. （The reason for exclusion: Irrelevant study outcome）

141. 孟令云, *房颤射频消融术后患者心脏结构与功能的改变*. 2020. （The reason for exclusion: Irrelevant study outcome）

142. 刘湘, et al., *心血管疾病合并心房颤动与慢性肺疾病合并心房颤动患者的临床特征研究 %J 中国全科医学.* 2020. **23**(26): p. 3274-3279. （The reason for exclusion: Irrelevant study outcome）

143. 李萌, *超声心动图评价阵发性心房颤动患者冷冻球囊消融术后复发的预测因子*. 2020. （The reason for exclusion: Irrelevant study outcome）

144. 李恋晨, *实时三维超声心动图评价慢性房颤患者右心房功能的研究*. 2020. （The reason for exclusion: Irrelevant study outcome）

145. 陈红霞, *左室右房通道与心房颤动的相关性研究*. 2020. （The reason for exclusion: Irrelevant study outcome）

146. Zeljkovic, I., et al., *Atrial appendages' mechanics assessed by 3D transoesophageal echocardiography as predictors of atrial fibrillation recurrence after pulmonary vein isolation.* Ijc Heart & Vasculature, 2020. **31**: p. 8. （The reason for exclusion: Irrelevant study outcome）

147. Vroomen, M., et al., *Epicardial and Endocardial Validation of Conduction Block After Thoracoscopic Epicardial Ablation of Atrial Fibrillation.* Innovations: Technology and Techniques in Cardiothoracic and Vascular Surgery, 2020. **15**(6): p. 525-531. （The reason for exclusion: Irrelevant study outcome）

148. Nct, *Effect of Venous Cannulation on the Incidence of Atrial Fibrillation in Patients Undergoing Coronary Artery Bypass.* https://clinicaltrials.gov/ct2/show/NCT04641611, 2020. （The reason for exclusion: Irrelevant study outcome）

149. Minamisaka, T., et al., *Protracted impairment of left atrial compliance after cryoballoon ablation in recurrence-free patients with paroxysmal atrial fibrillation.* American Journal of Cardiovascular Disease, 2020. **10**(4): p. 514-521. （The reason for exclusion: Irrelevant study outcome）

150. Kamioka, M., et al., *The efficacy of combination of transcatheter atrial septal defects closure and radiofrequency catheter ablation for the prevention of atrial fibrillation recurrence through bi-atrial reverse remodeling.* J Interv Card Electrophysiol, 2020. **59**(2): p. 365-372. （The reason for exclusion: Irrelevant study outcome）

151. Kamioka, M., et al., *The efficacy of combination of transcatheter atrial septal defects closure and radiofrequency catheter ablation for the prevention of atrial fibrillation recurrence through bi-atrial reverse remodeling.* Journal of Interventional Cardiac Electrophysiology, 2020. **59**(2): p. 365-372. （The reason for exclusion: duplicate record）

152. Kamioka, M., et al., *The efficacy of combination of transcatheter atrial septal defects closure and radiofrequency catheter ablation for the prevention of atrial fibrillation recurrence through bi-atrial reverse remodeling.* Journal of Interventional Cardiac Electrophysiology, 2020. **59**(2): p. 365-372. （The reason for exclusion: duplicate record）

153. Guttman, M.A., et al., *Acute enhancement of necrotic radio-frequency ablation lesions in left atrium and pulmonary vein ostia in swine model with non-contrast-enhanced T(1) -weighted MRI.* Magn Reson Med, 2020. **83**(4): p. 1368-1379. （The reason for exclusion: animal experiment）

154. Guttman, M.A., et al., *Acute enhancement of necrotic radio-frequency ablation lesions in left atrium and pulmonary vein ostia in swine model with non-contrast-enhanced <i>T</i><sub>1</sub>-weighted MRI.* Magnetic Resonance in Medicine, 2020. **83**(4): p. 1368-1379. （The reason for exclusion: duplicate record）

155. Guttman, M.A., et al., *Acute enhancement of necrotic radio-frequency ablation lesions in left atrium and pulmonary vein ostia in swine model with non-contrast-enhanced T1-weighted MRI.* Magnetic Resonance in Medicine, 2020. **83**(4): p. 1368-1379. （The reason for exclusion: duplicate record）

156. Creta, A., et al., *Impact of Type-2 Diabetes Mellitus on the Outcomes of Catheter Ablation of Atrial Fibrillation (European Observational Multicentre Study).* American Journal of Cardiology, 2020. **125**(6): p. 901-906. （The reason for exclusion: Irrelevant study outcome）

157. Blîndu, E., et al., *Epicardial Fat Volume as a New Imaging-Based Feature Associated with Risk of Recurrence after Pulmonary Veins Ablation in Atrial Fibrillation.* Journal of Interdisciplinary Medicine, 2020. **5**(2): p. 65-70. （The reason for exclusion: Irrelevant study outcome）

158. 叶坤, *急慢性阻塞性睡眠呼吸暂停相关心房颤动犬模型心脏结构重构的研究*. 2019. （The reason for exclusion: animal experiment）

159. 齐琳, *持续性房颤患者射频消融术后复发的超声心动图相关因素评价*. 2019. （The reason for exclusion: Irrelevant study outcome）

160. 梅帆 and 聂钰君, *风湿性心脏病伴长RR间期的心内电生理研究 %J 中国继续医学教育.* 2019. **11**(12): p. 93-96. （The reason for exclusion: Irrelevant study outcome）

161. 黄楚翘, *房颤合并功能性三尖瓣反流的右心超声心动图改变及其机制探讨*. 2019. （The reason for exclusion: Irrelevant study outcome）

162. 樊雪, *超声心动图评价房颤射频消融不同手术方式对左心房功能的影响*. 2019. （The reason for exclusion: Irrelevant study outcome）

163. Zeljkovic, I., et al., *Atrial appendage mechanics and superior vena cava area assessed by transoesophageal echocardiography in prediction of atrial fibrillation recurrence after pulmonary vein isolation.* Europace, 2019. **21**: p. ii400. （The reason for exclusion: Irrelevant study outcome）

164. Yan, Y. and X.L. Li, *[Evaluation of Left Atrial Structure and Function with Two-dimensional Speckle Tracking Imaging and Real-time Three-dimensional Imaging in Patients with Paroxysmal Atrial Fibrillation After Radiofrequency Catheter Ablation].* Sichuan Da Xue Xue Bao Yi Xue Ban, 2019. **50**(3): p. 390-395. （The reason for exclusion: Irrelevant study outcome）

165. Yan, Y. and X.L. Li, *Evaluation of Left Atrial Structure and Function with Two-dimensional Speckle Tracking Imaging and Real-time Three-dimensional Imaging in Patients with Paroxysmal Atrial Fibrillation After Radiofrequency Catheter Ablation.* Sichuan da xue xue bao. Yi xue ban = Journal of Sichuan University. Medical science edition, 2019. **50**(3): p. 390-395. （The reason for exclusion: Irrelevant study outcome）

166. Vroomen, M., et al., *Quantification of epicardial adipose tissue in patients undergoing hybrid ablation for atrial fibrillation.* Eur J Cardiothorac Surg, 2019. **56**(1): p. 79-86. （The reason for exclusion: Irrelevant study outcome）

167. Soulat-Dufour, L., et al., *Initial bi atrial three-dimensional echocardiographic evaluation in non-valvular atrial fibrillation according to rhythm outcome at six month follow-up.* Archives of Cardiovascular Diseases Supplements, 2019. **11**(1): p. 55. （The reason for exclusion: Irrelevant study outcome）

168. Santoro, F., et al., *Second-Generation Cryoballoon Atrial Fibrillation Ablation in Patients With Persistent Left Superior Caval Vein.* Jacc-Clinical Electrophysiology, 2019. **5**(5): p. 590-598. （The reason for exclusion: Irrelevant study outcome）

169. Santoro, F., et al., *Second-Generation Cryoballoon Atrial Fibrillation Ablation in Patients With Persistent Left Superior Caval Vein.* JACC Clin Electrophysiol, 2019. **5**(5): p. 590-598. （The reason for exclusion: duplicate record）

170. Khine, S., et al., *Cryoablation for paroxysmal atrial fibrillation: Procedure success and recurrence rate at 12 months follow-up.* Journal of Arrhythmia, 2019. **35**: p. 295. （The reason for exclusion: Irrelevant study outcome）

171. Kamioka, M., et al., *Combination impact of transcatheter atrial septal defects closure and radiofrequency catheter ablation on atrial fibrillation recurrence through bi-atrial reverse remodeling.* Journal of Arrhythmia, 2019. **35**: p. 113. （The reason for exclusion: Irrelevant study outcome）

172. Johner, N., et al., *Evolution of post-pulmonary vein isolation atrial fibrillation inducibility at redo ablation: Electrophysiological evidence of extra-pulmonary vein substrate progression.* Heart Rhythm, 2019. **16**(8): p. 1160-1166. （The reason for exclusion: Irrelevant study outcome）

173. Istratoaie, S., et al., *The Impact of Pulmonary Vein Anatomy on the Outcomes of Catheter Ablation for Atrial Fibrillation.* Medicina-Lithuania, 2019. **55**(11): p. 10. （The reason for exclusion: Irrelevant study outcome）

174. Istratoaie, S., et al., *The Impact of Pulmonary Vein Anatomy on the Outcomes of Catheter Ablation for Atrial Fibrillation.* Medicina (Kaunas), 2019. **55**(11). （The reason for exclusion: duplicate record）

175. Istratoaie, S., et al., *The Impact of Pulmonary Vein Anatomy on the Outcomes of Catheter Ablation for Atrial Fibrillation.* Medicina (Kaunas, Lithuania), 2019. **55**(11). （The reason for exclusion: duplicate record）

176. Ipek, E.G., et al., *Predictors and Incidence of Atrial Flutter After Catheter Ablation of Atrial Fibrillation.* American Journal of Cardiology, 2019. **124**(11): p. 1690-1696. （The reason for exclusion: Irrelevant study outcome）

177. Gucuk Ipek, E., et al., *Predictors and Incidence of Atrial Flutter After Catheter Ablation of Atrial Fibrillation.* Am J Cardiol, 2019. **124**(11): p. 1690-1696. （The reason for exclusion: Irrelevant study outcome）

178. Gucuk Ipek, E., et al., *Predictors and Incidence of Atrial Flutter After Catheter Ablation of Atrial Fibrillation.* American Journal of Cardiology, 2019. **124**(11): p. 1690-1696. （The reason for exclusion: duplicate record）

179. Garvanski, I., et al., *Predictors of Recurrence of AF in Patients After Radiofrequency Ablation.* European Cardiology Review, 2019. **14**(3): p. 165-168. （The reason for exclusion: without relevant date）

180. Fujimoto, Y., et al., *New Electrocardiographic Marker as “Notched P Wave Component” for Recurrence of Atrial Fibrillation after Electrical Cardioversion.* Journal of Electrocardiology, 2019. **53**: p. e5. （The reason for exclusion: Irrelevant study outcome）

181. Bordi, L., et al., *The influence of right atrial volume on atrial fibrillation reccurence.* European Heart Journal Cardiovascular Imaging, 2019. **20**: p. i72. （The reason for exclusion: without relevant date）

182. 欧阳书堃 and 肖骅, *房颤导管消融术后复发和预测因素研究进展 %J 现代医药卫生.* 2018. **34**(19): p. 3014-3018. （The reason for exclusion: review）

183. 郝志宏, *cGMP/Akt/GSK-3β信号途径对快速心房起搏家兔ANP分泌的调节作用*. 2018. （The reason for exclusion: animal experiment）

184. Yamaguchi, N., et al., *Impact of Sinus Node Recovery Time after Long-Standing Atrial Fibrillation Termination on the Long-Term Outcome of Catheter Ablation.* Int Heart J, 2018. **59**(3): p. 497-502. （The reason for exclusion: Irrelevant study outcome）

185. Yamaguchi, N., et al., *Impact of sinus node recovery time after long-standing atrial fibrillation termination on the long-term outcome of catheter ablation.* International Heart Journal, 2018. **59**(3): p. 497-502. （The reason for exclusion: duplicate record）

186. Yamaguchi, N., et al., *Impact of Sinus Node Recovery Time after Long-Standing Atrial Fibrillation Termination on the Long-Term Outcome of Catheter Ablation.* International Heart Journal, 2018. **59**(3): p. 497-502. （The reason for exclusion: duplicate record）

187. Vandenberk, B., et al., *Clinical predictors of success of radiofrequency pulmonary vein isolation.* Acta Cardiologica, 2018. **73**(5): p. 504-505. （The reason for exclusion: Irrelevant study outcome）

188. Nct, *Changes in Cardiac Autonomic Nervous System Following Atrial Fibrillation Ablation.* https://clinicaltrials.gov/show/NCT03811639, 2018. （The reason for exclusion: Irrelevant study outcome）

189. Nct, *Cryoballoon Pulmonary Vein Isolation vs. Cryoballoon Pulmonary Vein Isolation With Additional Right Atrial Linear Ablation for Persistent Atrial Fibrillation (CRARAL Trial).* https://clinicaltrials.gov/show/NCT03682887, 2018. （The reason for exclusion: Irrelevant study outcome）

190. Nakashima, T., et al., *Impact of the pulmonary vein orifice area assessed using intracardiac echocardiography on the outcome of radiofrequency catheter ablation for atrial fibrillation.* Journal of Interventional Cardiac Electrophysiology, 2018. **51**(2): p. 133-142. （The reason for exclusion: Irrelevant study outcome）

191. Maeda, M., et al., *Usefulness of Epicardial Adipose Tissue Volume to Predict Recurrent Atrial Fibrillation After Radiofrequency Catheter Ablation.* American Journal of Cardiology, 2018. **122**(10): p. 1694-1700. （The reason for exclusion: Irrelevant study outcome）

192. Kumagai, Y., et al., *Biatrial volume, estimated using magnetic resonance imaging, predicts atrial fibrillation recurrence after ablation.* Pacing Clin Electrophysiol, 2018. **41**(12): p. 1635-1642. （This document meets the inclusion criteria）

193. Kumagai, Y., et al., *Biatrial volume, estimated using magnetic resonance imaging, predicts atrial fibrillation recurrence after ablation.* PACE - Pacing and Clinical Electrophysiology, 2018. **41**(12): p. 1635-1642. （The reason for exclusion: duplicate record）

194. Kim, D., et al., *Sinus node dysfunction after surgical atrial fibrillation ablation with concomitant mitral valve surgery: Determinants and clinical outcomes.* PLoS One, 2018. **13**(9): p. e0203828. （The reason for exclusion: Irrelevant study outcome）

195. Kaypakli, O., et al., *Association of P wave duration index with atrial fibrillation recurrence after cryoballoon catheter ablation.* Journal of Electrocardiology, 2018. **51**(2): p. 182-187. （The reason for exclusion: Irrelevant study outcome）

196. Garweg, C., et al., *High-Detailed evaluation of the right atrial anatomy by three-dimensional rotational angiography during ablation procedures for atrioventricular nodal reentrant tachycardia and atrial flutter.* Scand Cardiovasc J, 2018. **52**(5): p. 268-274. （The reason for exclusion: Irrelevant study outcome）

197. Fujimoto, Y., et al., *Advanced interatrial block is an electrocardiographic marker for recurrence of atrial fibrillation after electrical cardioversion.* Int J Cardiol, 2018. **272**: p. 113-117. The reason for exclusion: Irrelevant study outcome）

198. Fujimoto, Y., et al., *Advanced interatrial block is an electrocardiographic marker for recurrence of atrial fibrillation after electrical cardioversion.* International Journal of Cardiology, 2018. **272**: p. 113-117. （The reason for exclusion: duplicate record）

199. Fujimoto, Y., et al., *Advanced interatrial block is an electrocardiographic marker for recurrence of atrial fibrillation after electrical cardioversion.* International Journal of Cardiology, 2018. **272**: p. 113-117. （The reason for exclusion: duplicate record）

200. Fujimoto, Y., et al., *Prediction of very late recurrence of atrial arrhythmias after an initial atrial fibrillation ablation session.* Circulation, 2018. **138**. （The reason for exclusion: Irrelevant study outcome）

201. Brynza, M., et al., *[FUNCTIONAL PARAMETERS OF BLOOD CIRCULATION IN FIRST THREE MONTHS AFTER RADIOFREQUENCY ABLATION OF ATRIAL FIBRILLATION AND FLUTTER].* Georgian Med News, 2018(279): p. 73-79. （The reason for exclusion: Irrelevant study outcome）

202. Brynza, M., et al., *FUNCTIONAL PARAMETERS OF BLOOD CIRCULATION IN FIRST THREE MONTHS AFTER RADIOFREQUENCY ABLATION OF ATRIAL FIBRILLATION AND FLUTTER.* Georgian medical news, 2018(279): p. 73-79. （The reason for exclusion: duplicate record）

203. Bai, Y., et al., *Association of peak atrial longitudinal strain with atrial fibrillation recurrence in patients with chronic lung diseases following radiofrequency ablation.* Internal Medicine Journal, 2018. **48**(7): p. 851-859. （The reason for exclusion: Irrelevant study outcome）

204. 苑晓倩, *三维斑点追踪成像技术评价病态窦房结综合征患者心房重构和电重构*. 2017. （The reason for exclusion: Irrelevant study outcome）

205. 郁怡, et al., *右房容积指数在射频消融手术联合心腔内电复律治疗持续性心房颤动及其远期复发中的应用价值 %J 中国超声医学杂志.* 2017. **33**(03): p. 220-224. （The reason for exclusion: without relevant date）

206. 马新欣, *二维斑点追踪成像技术对房颤消融术后远期复发的预测价值*. 2017. （The reason for exclusion: Irrelevant study outcome）

207. 刘魁智 and 邱春光, *左右心房在心房颤动复发方面影响的探究 %J 中国实用医刊 %J Chinese Journal of Practical Medicine.* 2017. **44**(12): p. 116-119. （The reason for exclusion: review）

208. 龚嘉淼, *三尖瓣反流对消融术后房颤复发的影响的研究*. 2017. （The reason for exclusion: review）

209. Yang, C.H., et al., *Comparisons of the underlying mechanisms of left atrial remodeling after repeat circumferential pulmonary vein isolation with or without additional left atrial linear ablation in patients with recurrent atrial fibrillation.* International Journal of Cardiology, 2017. **228**: p. 449-455. （The reason for exclusion: Irrelevant study outcome）

210. Tiano, J.J., et al., *Transvenous Before Surgical Hybrid Procedure.* J La State Med Soc, 2017. **169**(3): p. 71-77. （The reason for exclusion: Irrelevant study outcome）

211. Makowski, M., et al., *Platelet reactivity and mean platelet volume as risk markers of thrombogenesis in atrial fibrillation.* International Journal of Cardiology, 2017. **235**: p. 1-5. （The reason for exclusion: Irrelevant study outcome）

212. Kim, I.S., et al., *Minimal energy requirement for external cardioversion and catheter ablation for long-standing persistent atrial fibrillation.* Journal of Cardiology, 2017. **69**(1-2): p. 162-168. （The reason for exclusion: Irrelevant study outcome）

213. Kim, D.H., S.W. Park, and Y.H. Kim, *Patients with thin left atrial wall thickness have a risk of severe left atrial volume reduction after atrial fibrillation ablation.* European Heart Journal Cardiovascular Imaging, 2017. **18**: p. i37-i38. （The reason for exclusion: duplicate record）

214. Berte, B., et al., *A new cryoenergy for ventricular tachycardia ablation: a proof-of-concept study.* Europace, 2017. **19**(8): p. 1401-1407. （The reason for exclusion: animal experiment）

215. Berte, B., et al., *A new cryoenergy for ventricular tachycardia ablation: a proof-of-concept study.* Europace, 2017. **19**(8): p. 1401-1407. （The reason for exclusion: duplicate record）

216. Berte, B., et al., *A new cryoenergy for ventricular tachycardia ablation: A proof-of-concept study.* Europace, 2017. **19**(8): p. 1401-1407. （The reason for exclusion: duplicate record）

217. Aryana, A., et al., *5-Year outcomes of a staged hybrid surgical and catheter-based ablation approach for treatment of long-standing persistent atrial fibrillation.* Journal of the American College of Cardiology, 2017. **69**(11): p. 449. （The reason for exclusion: Irrelevant study outcome）

218. 郁怡, et al. *右房容积指数在射频消融手术联合心腔内电复律治疗持续性心房颤动及其远期复发中的应用价值*. in *中国超声医学工程学会第十三届全国超声心动图学术会议*. 2016. 中国北京. （The reason for exclusion: meeting abstract）

219. 闻松男, et al., *右心房大小不能预测持续性心房颤动导管射频消融术后的复发 %J 心肺血管病杂志.* 2016. **35**(09): p. 686-689. （The reason for exclusion: without relevant date）

220. 闻松男, et al., *右心房大小预测阵发性心房颤动合并左心房增大导管射频消融术后的复发 %J 临床心血管病杂志.* 2016. **32**(08): p. 794-798. （The reason for exclusion: duplicate record）

221. 沈下贤, et al., *慢性心力衰竭患者心脏再同步化治疗的疗效及其影响因素分析 %J 现代生物医学进展.* 2016. **16**(18): p. 3460-3463. （The reason for exclusion: Irrelevant study outcome）

222. Sasaki, T., et al., *The Right to Left Atrial Volume Ratio Predicts Outcomes after Circumferential Pulmonary Vein Isolation of Longstanding Persistent Atrial Fibrillation.* Pacing Clin Electrophysiol, 2016. **39**(11): p. 1181-1190. （The reason for exclusion: without relevant date）

223. Sasaki, T., et al., *The Right to Left Atrial Volume Ratio Predicts Outcomes after Circumferential Pulmonary Vein Isolation of Longstanding Persistent Atrial Fibrillation.* Pace-Pacing and Clinical Electrophysiology, 2016. **39**(11): p. 1181-1190. （The reason for exclusion: duplicate record）

224. Okada, A., et al., *The recurrence of atrial fibrillation was associated with high oxidative stress in coronary sinus vein.* Circulation, 2016. **134**. （The reason for exclusion: Irrelevant study outcome）

225. Masuda, M., et al., *Steerable versus non-steerable sheaths during pulmonary vein isolation: impact of left atrial enlargement on the catheter-tissue contact force.* J Interv Card Electrophysiol, 2016. **47**(1): p. 99-107. （The reason for exclusion: Irrelevant study outcome）

226. Luong, C.L., et al., *Usefulness of the Atrial Emptying Fraction to Predict Maintenance of Sinus Rhythm After Direct Current Cardioversion for Atrial Fibrillation.* Am J Cardiol, 2016. **118**(9): p. 1345-1349. （The reason for exclusion: Irrelevant study outcome）

227. Lee, A., et al., *Atrial fibrillation ablation by single ring isolation versus wide antral isolation: Effects on left atrial size and function.* International Journal of Cardiology, 2016. **206**: p. 1-6. （The reason for exclusion: Irrelevant study outcome）

228. Chrispin, J., et al., *Lack of regional association between atrial late gadolinium enhancement on cardiac magnetic resonance and atrial fibrillation rotors.* Heart Rhythm, 2016. **13**(3): p. 654-60. （The reason for exclusion: Irrelevant study outcome）

229. Buist, T.J., et al., *Association between pulmonary vein orientation and ablation outcome in patients undergoing multi-electrode ablation for atrial fibrillation.* Journal of Cardiovascular Computed Tomography, 2016. **10**(3): p. 251-257. （The reason for exclusion: Irrelevant study outcome）

230. 吴起才, et al., *心内直视手术同期双极射频消融治疗心房颤动137例的临床效果 %J 中国胸心血管外科临床杂志.* 2015. **22**(09): p. 807-811. （The reason for exclusion: Irrelevant study outcome）

231. 刘颖娴, et al., *心房颤动患者右心结构及压力变化研究 %J 中国心血管杂志.* 2015. **20**(01): p. 34-38. （The reason for exclusion: Irrelevant study outcome）

232. Sramko, M., et al., *Clinical value of assessment of left atrial late gadolinium enhancement in patients undergoing ablation of atrial fibrillation.* International Journal of Cardiology, 2015. **179**: p. 351-357. （The reason for exclusion: Irrelevant study outcome）

233. Sotomi, Y., et al., *Persistent left atrial remodeling after catheter ablation for non-paroxysmal atrial fibrillation is associated with very late recurrence.* Journal of Cardiology, 2015. **66**(5-6): p. 370-376. （The reason for exclusion: Irrelevant study outcome）

234. Schluermann, F., et al., *<i>In vivo</i> contact force measurements and correlation with left atrial anatomy during catheter ablation of atrial fibrillation.* Europace, 2015. **17**(10): p. 1526-1532. （The reason for exclusion: Irrelevant study outcome）

235. Rettmann, M.E., et al., *Measurements of the left atrium and pulmonary veins for analysis of reverse structural remodeling following cardiac ablation therapy.* Computer Methods and Programs in Biomedicine, 2015. **118**(2): p. 198-206. （The reason for exclusion: Irrelevant study outcome）

236. Pontone, G., et al., *Comparison of cardiac computed tomography versus cardiac magnetic resonance for characterization of left atrium anatomy before radiofrequency catheter ablation of atrial fibrillation.* International Journal of Cardiology, 2015. **179**: p. 114-121. （The reason for exclusion: Irrelevant study outcome）

237. Nakatani, Y., et al., *Location of epicardial adipose tissue affects the efficacy of a combined dominant frequency and complex fractionated atrial electrogram ablation of atrial fibrillation.* Heart Rhythm, 2015. **12**(2): p. 257-65. （The reason for exclusion: Irrelevant study outcome）

238. Moon, J., et al., *Prognostic Implications of Right and Left Atrial Enlargement after Radiofrequency Catheter Ablation in Patients with Nonvalvular Atrial Fibrillation.* Korean Circ J, 2015. **45**(4): p. 301-9. (This document meets the inclusion criteria)

239. Moon, J., et al., *Prognostic Implications of Right and Left Atrial Enlargement after Radiofrequency Catheter Ablation in Patients with Nonvalvular Atrial Fibrillation.* Korean Circulation Journal, 2015. **45**(4): p. 301-309. （The reason for exclusion: duplicate record）

240. Moon, J., et al., *Prognostic implications of right and left atrial enlargement after radiofrequency catheter ablation in patients with nonvalvular atrial fibrillation.* Korean Circulation Journal, 2015. **45**(4): p. 301-309. （The reason for exclusion: duplicate record）

241. Luong, C., et al., *Right atrial volume is superior to left atrial volume for prediction of atrial fibrillation recurrence after direct current cardioversion.* Can J Cardiol, 2015. **31**(1): p. 29-35. (This document meets the inclusion criteria)

242. Loardi, C., et al., *Surgical Treatment of Concomitant Atrial Fibrillation: Focus onto Atrial Contractility.* Biomed Res Int, 2015. **2015**: p. 274817. （The reason for exclusion: Irrelevant study outcome）

243. Loardi, C., et al., *Surgical treatment of concomitant atrial fibrillation: Focus onto atrial contractility.* BioMed Research International, 2015. **2015**. （The reason for exclusion: duplicate record）

244. Loardi, C., et al., *Surgical Treatment of Concomitant Atrial Fibrillation: Focus onto Atrial Contractility.* Biomed Research International, 2015. **2015**: p. 9. （The reason for exclusion: duplicate record）

245. Kocyigit, D., et al., *Periatrial epicardial adipose tissue thickness is an independent predictor of atrial fibrillation recurrence after cryoballoon-based pulmonary vein isolation.* Journal of Cardiovascular Computed Tomography, 2015. **9**(4): p. 295-302. （The reason for exclusion: Irrelevant study outcome）

246. Kim, T.H., et al., *Blunted rate-dependent left atrial pressure response during isoproterenol infusion in atrial fibrillation patients with impaired left ventricular diastolic function: a comparison to pacing.* Europace, 2015. **17**: p. 89-96. （The reason for exclusion: Irrelevant study outcome）

247. Kim, J.S., et al., *Does isolation of the left atrial posterior wall improve clinical outcomes after radiofrequency catheter ablation for persistent atrial fibrillation? A prospective randomized clinical trial.* International Journal of Cardiology, 2015. **181**: p. 277-283. （The reason for exclusion: Irrelevant study outcome）

248. Hanazawa, K., et al., *Effect of radiofrequency catheter ablation of persistent atrial fibrillation on the left atrial function: Assessment by 320-row multislice computed tomography.* International Journal of Cardiology, 2015. **179**: p. 449-454. （The reason for exclusion: Irrelevant study outcome）

249. Güler, E., et al., *Effect of Pulmonary Vein Anatomy and Pulmonary Vein Diameters on Outcome of Cryoballoon Catheter Ablation for Atrial Fibrillation.* Pacing Clin Electrophysiol, 2015. **38**(8): p. 989-96. （The reason for exclusion: Irrelevant study outcome）

250. Gal, P., et al., *Association between pulmonary vein orientation and atrial fibrillation-free survival in patients undergoing endoscopic laser balloon ablation.* European Heart Journal-Cardiovascular Imaging, 2015. **16**(7): p. 799-806. （The reason for exclusion: Irrelevant study outcome）

251. Canpolat, U., et al., *The impact of cryoballoon-based catheter ablation on left atrial structural and potential electrical remodeling in patients with paroxysmal atrial fibrillation.* J Interv Card Electrophysiol, 2015. **44**(2): p. 131-9. （The reason for exclusion: Irrelevant study outcome）

252. Canpolat, U., et al., *The impact of cryoballoon-based catheter ablation on left atrial structural and potential electrical remodeling in patients with paroxysmal atrial fibrillation.* Journal of Interventional Cardiac Electrophysiology, 2015. **44**(2): p. 131-139. （The reason for exclusion: duplicate record）

253. Canpolat, U., et al., *The impact of cryoballoon-based catheter ablation on left atrial structural and potential electrical remodeling in patients with paroxysmal atrial fibrillation.* Journal of interventional cardiac electrophysiology, 2015. **44**(2): p. 131‐139. （The reason for exclusion: duplicate record）

254. Canpolat, U., et al., *The impact of cryoballoon-based catheter ablation on left atrial structural and potential electrical remodeling in patients with paroxysmal atrial fibrillation.* Journal of Interventional Cardiac Electrophysiology, 2015. **44**(2): p. 131-139. （The reason for exclusion: duplicate record）

255. Abo-Salem, E., et al., *Surgical Treatment of Atrial Fibrillation.* J Cardiovasc Electrophysiol, 2015. **26**(9): p. 1027-1037. （The reason for exclusion: review）

256. Abo-Salem, E., et al., *Surgical Treatment of Atrial Fibrillation.* Journal of Cardiovascular Electrophysiology, 2015. **26**(9): p. 1027-1037. （The reason for exclusion: duplicate record）

257. Abo-Salem, E., et al., *Surgical treatment of atrial fibrillation.* Journal of Cardiovascular Electrophysiology, 2015. **26**(9): p. 1027-1037. （The reason for exclusion: duplicate record）

258. 吴绍辉, et al., *已达到消融终点的长程持续性心房颤动复发危险因素分析 %J 国际心血管病杂志 %J International Journal of Cardiovascular Disease.* 2014(4): p. 268-270. （The reason for exclusion: review）

259. 王茜, et al., *心房追踪技术对心房颤动患者右心房功能的评价 %J 中国心血管杂志.* 2014. **19**(01): p. 12-15. （The reason for exclusion: Irrelevant study outcome）

260. 王茜, *超声心动图对心房颤动者心功能的评价*. 2014. （The reason for exclusion: duplicate record）

261. 巩江华, *实时三维超声及斑点追踪技术评价房性心律失常患者心房功能的研究*. 2014. （The reason for exclusion: Irrelevant study outcome）

262. Kim, T.H., et al., *Pericardial fat volume is associated with clinical recurrence after catheter ablation for persistent atrial fibrillation, but not paroxysmal atrial fibrillation: An analysis of over 600-patients.* International Journal of Cardiology, 2014. **176**(3): p. 841-846. （The reason for exclusion: Irrelevant study outcome）

263. Hirai, T., et al., *Usefulness of left ventricular diastolic function to predict recurrence of atrial fibrillation in patients with preserved left ventricular systolic function.* American Journal of Cardiology, 2014. **114**(1): p. 65-69. （The reason for exclusion: Irrelevant study outcome）

264. Han, S.W., et al., *Does the amount of atrial mass reduction improve clinical outcomes after radiofrequency catheter ablation for long-standing persistent atrial fibrillation? Comparison between linear ablation and defragmentation.* International Journal of Cardiology, 2014. **171**(1): p. 37-43. （The reason for exclusion: Irrelevant study outcome）

265. Gebhard, C., et al., *Characterization of Pulmonary Vein Dimensions Using High-Definition 64-Slice Computed Tomography prior to Radiofrequency Catheter Ablation for Atrial Fibrillation.* Cardiol Res Pract, 2014. **2014**: p. 179632. （The reason for exclusion: Irrelevant study outcome）

266. Gebhard, C., et al., *Characterization of pulmonary vein dimensions using high-definition 64-slice computed tomography prior to radiofrequency catheter ablation for atrial fibrillation.* Cardiology Research and Practice, 2014. **2014**. （The reason for exclusion: duplicate record）

267. Ejima, K., et al., *Long-term outcome and preprocedural predictors of atrial tachyarrhythmia recurrence following pulmonary vein antrum isolation-based catheter ablation in patients with non-paroxysmal atrial fibrillation.* J Cardiol, 2014. **64**(1): p. 57-63. （The reason for exclusion: Irrelevant study outcome）

268. Cabanas Grandio, P., et al., *MRI characterization of cryoballoon and radiofrequency ablation lesions after pulmonary vein isolation.* European Heart Journal, 2014. **35**: p. 428. （The reason for exclusion: Irrelevant study outcome）

269. Zhao, L., et al., *Why atrial fibrillation recurs in patients who obtained current ablation endpoints with longstanding persistent atrial fibrillation.* Journal of Interventional Cardiac Electrophysiology, 2013. **37**(3): p. 283-290. (This document meets the inclusion criteria)

270. Yorgun, H., et al., *Successful cryoballoon ablation of paroxysmal atrial fibrillation ensures left atrial structural and electrical reverse remodelling.* International Journal of Cardiology, 2013. **163**(3): p. S75-S76. （The reason for exclusion: Irrelevant study outcome）

271. Uhm, J.S., et al., *Prolonged Atrial Effective Refractory Periods in Atrial Fibrillation Patients Associated with Structural Heart Disease or Sinus Node Dysfunction Compared with Lone Atrial Fibrillation.* Pace-Pacing and Clinical Electrophysiology, 2013. **36**(2): p. 163-171. （The reason for exclusion: Irrelevant study outcome）

272. Silva-Palacios, F., et al., *Recurrence of atrial fibrillation after radiofrequency ablation is associated with peri-pulmonary vein fat measured by non gated computed tomography.* Journal of Cardiovascular Computed Tomography, 2013. **7**: p. S33-S34. （The reason for exclusion: Irrelevant study outcome）

273. Shim, J., et al., *Long duration of radiofrequency energy delivery is an independent predictor of clinical recurrence after catheter ablation of atrial fibrillation: Over 500 cases experience.* International Journal of Cardiology, 2013. **167**(6): p. 2667-2672. （The reason for exclusion: Irrelevant study outcome）

274. Park, J., et al., *Post-shock sinus node recovery time is an independent predictor of recurrence after catheter ablation of longstanding persistent atrial fibrillation.* International Journal of Cardiology, 2013. **168**(3): p. 1937-1942. （The reason for exclusion: Irrelevant study outcome）

275. Neilan, T.G., et al., *Effect of sleep apnea and continuous positive airway pressure on cardiac structure and recurrence of atrial fibrillation.* J Am Heart Assoc, 2013. **2**(6): p. e000421. （The reason for exclusion: Irrelevant study outcome）

276. Moon, J., et al., *Distinct prognostic impacts of both atrial volumes on outcomes after radiofrequency ablation of nonvalvular atrial fibrillation: three-dimensional imaging study using multidetector computed tomography.* Int J Cardiol, 2013. **168**(6): p. 5430-6. (This document meets the inclusion criteria)

277. Moon, J., et al., *Distinct prognostic impacts of both atrial volumes on outcomes after radiofrequency ablation of nonvalvular atrial fibrillation: Three-dimensional imaging study using multidetector computed tomography.* International Journal of Cardiology, 2013. **168**(6): p. 5430-5436. （The reason for exclusion: duplicate record）

278. Moon, J., et al., *Distinct prognostic implications of atrial anatomical remodeling after radiofrequency ablation between paroxysmal and persistent atrial fibrillation.* International Journal of Cardiology, 2013. **164**(2): p. S10. （The reason for exclusion: without relevant date）

279. Moon, J., et al., *Distinct prognostic implications of at rial anatomical remodeling after radiofrequency ablation between paroxysmal and persistent at rial fibrillation: 3d imaging study using multidetector computed tomography.* Journal of the American College of Cardiology, 2013. **61**(10): p. E366. （The reason for exclusion: duplicate record）

280. Moon, J., et al., *Prognostic implication of atrial pressures in patients with nonvalvular atrial fibrillation and preserved systolic function who undergo radiofrequency catheter ablation: A pilot study.* Circulation, 2013. **128**(22). （The reason for exclusion: Irrelevant study outcome）

281. Lo, L.W., et al., *Differences in the atrial electrophysiological properties between vagal and sympathetic types of atrial fibrillation.* J Cardiovasc Electrophysiol, 2013. **24**(6): p. 609-16. （The reason for exclusion: Irrelevant study outcome）

282. Lina, H. and Z. Shulong, *The impact of volume of pulmonary veins antrum and left atrium on recurrence of atrial fibrillation after radiofrequency catheter ablation in a mid and short-term period.* Heart, 2013. **99**: p. A5. （The reason for exclusion: Irrelevant study outcome）

283. Limantoro, I., et al., *Low efficacy of cardioversion of persistent atrial fibrillation with the implantable cardioverter-defibrillator.* Netherlands Heart Journal, 2013. **21**(12): p. 548-553. （The reason for exclusion: Irrelevant study outcome）

284. Jones, D.G., et al., *Impact of stepwise ablation on the biatrial substrate in patients with persistent atrial fibrillation and heart failure.* Circ Arrhythm Electrophysiol, 2013. **6**(4): p. 761-8. （The reason for exclusion: Irrelevant study outcome）

285. Ito, Y., et al., *Effect of Eplerenone on Maintenance of Sinus Rhythm After Catheter Ablation in Patients With Long-Standing Persistent Atrial Fibrillation.* American Journal of Cardiology, 2013. **111**(7): p. 1012-1018. （The reason for exclusion: Irrelevant study outcome）

286. Hakalahti, A., et al., *Diastolic echocardiographic parameters in predicting outcome of radiofrequency catheter ablation of atrial fibrillation.* Open Pacing, Electrophysiology and Therapy Journal, 2013. **5**: p. 1-5. （The reason for exclusion: without relevant date）

287. Chen, Y.L., et al., *The spatial distribution of atrial fibrillation termination sites in the right atrium during complex fractionated atrial electrograms-guided ablation in patients with persistent atrial fibrillation.* J Cardiovasc Electrophysiol, 2013. **24**(9): p. 949-57. （The reason for exclusion: Irrelevant study outcome）

288. 杨倩, *心耳尖部房速的特点及消融结果和肺静脉解剖与心房颤动的关系*. 2012, 清华大学医学部 北京协和医学院 中国医学科学院. （The reason for exclusion: Irrelevant study outcome）

289. 武长礼 and 肖学钧, *三尖瓣关闭不全与右心房重构关系研究进展 %J 岭南心血管病杂志.* 2012. **18**(03): p. 317-320. （The reason for exclusion: review）

290. Sponga, S., et al., *Role of an aggressive rhythm control strategy on sinus rhythm maintenance following intra-operative radiofrequency ablation of atrial fibrillation in patients undergoing surgical correction of valvular disease.* Journal of Cardiology, 2012. **60**(3-4): p. 316-320. （The reason for exclusion: Irrelevant study outcome）

291. Rettmann, M.E., et al., *Centerline Tracking for Quantification of Reverse Structural Remodeling of the Pulmonary Veins Following Cardiac Ablation Therapy.* Academic Radiology, 2012. **19**(11): p. 1332-1344. （The reason for exclusion: Irrelevant study outcome）

292. Park, Y.M., et al., *Is Pursuit of Termination of Atrial Fibrillation During Catheter Ablation of Great Value in Patients with Longstanding Persistent Atrial Fibrillation?* Journal of Cardiovascular Electrophysiology, 2012. **23**(10): p. 1051-1058. （The reason for exclusion: Irrelevant study outcome）

293. Park, J., et al., *Post-shock sinus node recovery time is an independent predictor of late recurrence after catheter ablation of persistent atrial fibrillation.* European Heart Journal, 2012. **33**: p. 986-987. （The reason for exclusion: Irrelevant study outcome）

294. Motooka, M., et al., *Association between atrial fibrillation recurrence and pulmonary vein contraction after radiofrequency catheter ablation: Assessment by 320-slice computed tomography.* European Heart Journal, 2012. **33**: p. 88. （The reason for exclusion: Irrelevant study outcome）

295. Morishima, I., et al., *Rescue pulmonaryvein isolation for hemodynamically unstable atrial fibrillation in a patient with an acute extensive myocardial infarction.* Heart Rhythm, 2012. **9**(5): p. S217-S218. （The reason for exclusion: Irrelevant study outcome）

296. Morishima, I., et al., *Rescue pulmonary vein isolation for hemodynamically unstable atrial fibrillation storm in a patient with an acute extensive myocardial infarction.* BMC Cardiovasc Disord, 2012. **12**: p. 110. （The reason for exclusion: duplicate record）

297. Morishima, I., et al., *Rescue pulmonary vein isolation for hemodynamically unstable atrial fibrillation storm in a patient with an acute extensive myocardial infarction.* Bmc Cardiovascular Disorders, 2012. **12**: p. 5. （The reason for exclusion: duplicate record）

298. Morishima, I., et al., *Rescue pulmonary vein isolation for hemodynamically unstable atrial fibrillation storm in a patient with an acute extensive myocardial infarction.* BMC Cardiovascular Disorders, 2012. **12**. （The reason for exclusion: duplicate record）

299. Moon, J., et al., *Right atrial anatomical remodeling affects early outcomes of nonvalvular atrial fibrillation after radiofrequency ablation.* Circ J, 2012. **76**(4): p. 860-7. (This document meets the inclusion criteria)

300. Moon, J., et al., *Right Atrial Anatomical Remodeling Affects Early Outcomes of Nonvalvular Atrial Fibrillation After Radiofrequency Ablation.* Circulation Journal, 2012. **76**(4): p. 860-867. （The reason for exclusion: duplicate record）

301. Moon, J., et al., *Right atrial anatomical remodeling affects early outcomes of nonvalvular atrial fibrillation after radiofrequency ablation.* Circulation Journal, 2012. **76**(4): p. 860-867. （The reason for exclusion: duplicate record）

302. Hartono, B., et al., *A novel finding of the atrial substrate properties and long-term results of catheter ablation in chronic atrial fibrillation patients with left atrial spontaneous echo contrast.* J Cardiovasc Electrophysiol, 2012. **23**(3): p. 239-46. （The reason for exclusion: Irrelevant study outcome）

303. Ari, H., et al., *A novel predictor of atrial fibrillation recurrence: Atrial electromechanical delay.* International Journal of Cardiology, 2012. **155**: p. S47-S48. （The reason for exclusion: Irrelevant study outcome）

304. Tsao, H.M., et al., *Quantitative analysis of quantity and distribution of epicardial adipose tissue surrounding the left atrium in patients with atrial fibrillation and effect of recurrence after ablation.* Am J Cardiol, 2011. **107**(10): p. 1498-503. （The reason for exclusion: Irrelevant study outcome）

305. Sohara, H., et al., *Long-term outcome following bi-atrial ablation using radio frequency hot balloon catheter for the patients with longstanding persistent atrial fibrillation.* Heart Rhythm, 2011. **8**(5): p. S178. （The reason for exclusion: Irrelevant study outcome）

306. Revishvili, A.S., et al., *Indications and results of surgical treatment of lone tachyarrhythmias.* Interactive Cardiovascular and Thoracic Surgery, 2011. **12**: p. S118. （The reason for exclusion: Irrelevant study outcome）

307. Montserrat, S., et al., *Effect of Repeated Radiofrequency Catheter Ablation on Left Atrial Function for the Treatment of Atrial Fibrillation.* American Journal of Cardiology, 2011. **108**(12): p. 1741-1746. （The reason for exclusion: Irrelevant study outcome）

308. Mazzetti, S., et al., *In patients with left ventricular dysfunction, Conversion of atrial fibrillation in sinus rhythm significantly changes the strain rate and the main echocardiography standard parameters.* European Journal of Heart Failure, Supplement, 2011. **10**: p. S16. （The reason for exclusion: Irrelevant study outcome）

309. Lo, L.W., et al., *Different patterns of atrial remodeling after catheter ablation of chronic atrial fibrillation.* J Cardiovasc Electrophysiol, 2011. **22**(4): p. 385-93. （The reason for exclusion: Irrelevant study outcome）

310. Lo, L.W., et al., *Different Patterns of Atrial Remodeling After Catheter Ablation of Chronic Atrial Fibrillation.* Journal of Cardiovascular Electrophysiology, 2011. **22**(4): p. 385-393. （The reason for exclusion: duplicate record）

311. Hof, I.E., et al., *Pulmonary vein antrum isolation leads to a significant decrease of left atrial size.* Europace, 2011. **13**(3): p. 371-5. （The reason for exclusion: Irrelevant study outcome）

312. Hof, I.E., et al., *Pulmonary vein antrum isolation leads to a significant decrease of left atrial size.* Europace, 2011. **13**(3): p. 371-375. （The reason for exclusion: duplicate record）

313. Hof, I.E., et al., *Pulmonary vein antrum isolation leads to a significant decrease of left atrial size.* Europace, 2011. **13**(3): p. 371-375. （The reason for exclusion: duplicate record）

314. den Uijl, D.W., et al., *Effect of Pulmonary Vein Anatomy and Left Atrial Dimensions on Outcome of Circumferential Radiofrequency Catheter Ablation for Atrial Fibrillation.* American Journal of Cardiology, 2011. **107**(2): p. 243-249. （The reason for exclusion: Irrelevant study outcome）

315. Caputo, M., et al., *Usefulness of Left Ventricular Diastolic Dysfunction Assessed by Pulsed Tissue Doppler Imaging as a Predictor of Atrial Fibrillation Recurrence After Successful Electrical Cardioversion.* American Journal of Cardiology, 2011. **108**(5): p. 698-704. （The reason for exclusion: Irrelevant study outcome）

316. Akutsu, Y., et al., *Association between left and right atrial remodeling with atrial fibrillation recurrence after pulmonary vein catheter ablation in patients with paroxysmal atrial fibrillation: a pilot study.* Circ Cardiovasc Imaging, 2011. **4**(5): p. 524-31. (This document meets the inclusion criteria)

317. 朱丽颖 and 程纯, *简述利钠肽与心房颤动的关系 %J 中国医药指南.* 2010. **8**(09): p. 48-51. （The reason for exclusion: review）

318. 甘天翊, et al., *心脏瓣膜病房颤患者左右心房纤维化机制比较 %J 中国现代医学杂志.* 2010. **20**(03): p. 395-399. （The reason for exclusion: Irrelevant study outcome）

319. Zhao, Q.Y., et al., *Atrial autonomic innervation remodelling and atrial fibrillation inducibility after epicardial ganglionic plexi ablation.* Europace, 2010. **12**(6): p. 805-10. （The reason for exclusion: Irrelevant study outcome）

320. Jeevanantham, V., et al., *Meta-Analysis of the Effect of Radiofrequency Catheter Ablation on Left Atrial Size, Volumes and Function in Patients With Atrial Fibrillation.* American Journal of Cardiology, 2010. **105**(9): p. 1317-1326. （The reason for exclusion: Irrelevant study outcome）

321. Chiu, F., et al., *Fractionated atrial electrograms in sinus rhythm predict outcome in patients with paroxysmal atrial fibrillation receiving pulmonary vein isolation by radiofrequency catheter ablation.* Heart Rhythm, 2010. **7**(5): p. S207. （The reason for exclusion: Irrelevant study outcome）

322. 许国军, et al., *慢性房颤患者左右心房胶原和基质金属蛋白酶表达差异 %J 生理学报.* 2009. **61**(03): p. 211-216. （The reason for exclusion: Irrelevant study outcome）

323. 程慧, et al., *心房颤动患者左心房内径与左心房血栓发生的关系 %J 中国循环杂志.* 2009. **24**(06): p. 451-453. （The reason for exclusion: Irrelevant study outcome）

324. 白文娟, et al., *心房颤动对左心瓣膜置换术后心脏重构的影响 %J 中华医学超声杂志(电子版).* 2009. **6**(02): p. 279-284. （The reason for exclusion: Irrelevant study outcome）

325. Stiles, M.K., et al., *Paroxysmal Lone Atrial Fibrillation Is Associated With an Abnormal Atrial Substrate.* Journal of the American College of Cardiology, 2009. **53**(14): p. 1182-1191. （The reason for exclusion: Irrelevant study outcome）

326. Peters, D.C., et al., *Recurrence of Atrial Fibrillation Correlates With the Extent of Post-Procedural Late Gadolinium Enhancement A Pilot Study.* Jacc-Cardiovascular Imaging, 2009. **2**(3): p. 308-316. （The reason for exclusion: Irrelevant study outcome）

327. Peters, D.C., et al., *Recurrence of atrial fibrillation correlates with the extent of post-procedural late gadolinium enhancement: a pilot study.* JACC Cardiovasc Imaging, 2009. **2**(3): p. 308-16. （The reason for exclusion: duplicate record）

328. Peters, D.C., et al., *Recurrence of Atrial Fibrillation Correlates With the Extent of Post-Procedural Late Gadolinium Enhancement. A Pilot Study.* JACC: Cardiovascular Imaging, 2009. **2**(3): p. 308-316. （The reason for exclusion: duplicate record）

329. Onorati, F., et al., *Results of Atrial Fibrillation Ablation during Mitral Surgery in Patients with Poor Electro-Anatomical Substrate.* Journal of Heart Valve Disease, 2009. **18**(6): p. 607-616. （The reason for exclusion: Irrelevant study outcome）

330. Choi, J.I., et al., *Clinical significance of complete conduction block of the left lateral isthmus and its relationship with anatomical variation of the vein of Marshall in patients with nonparoxysmal atrial fibrillation.* J Cardiovasc Electrophysiol, 2009. **20**(6): p. 616-22. （The reason for exclusion: Irrelevant study outcome）

331. 白文娟 and 唐红. *心房颤动对左心瓣膜置换术后心脏重构的影响*. in *庆祝中国超声诊断50年暨第十届全国超声医学学术会议*. 2008. 中国北京. （The reason for exclusion: meeting abstract）

332. Shin, S.H., et al., *Left atrial volume is a predictor of atrial fibrillation recurrence after catheter ablation.* J Am Soc Echocardiogr, 2008. **21**(6): p. 697-702. （The reason for exclusion: Irrelevant study outcome）

333. Shin, S.H., et al., *Left atrial volume is a predictor of atrial fibrillation recurrence after catheter ablation.* Journal of the American Society of Echocardiography, 2008. **21**(6): p. 697-702. （The reason for exclusion: duplicate record）

334. Shin, S.H., et al., *Left Atrial Volume Is a Predictor of Atrial Fibrillation Recurrence After Catheter Ablation.* Journal of the American Society of Echocardiography, 2008. **21**(6): p. 697-702. （The reason for exclusion: duplicate record）

335. Müller, H., et al., *Biatrial anatomical reverse remodelling after radiofrequency catheter ablation for atrial fibrillation: evidence from real-time three-dimensional echocardiography.* Europace, 2008. **10**(9): p. 1073-8. （The reason for exclusion: Irrelevant study outcome）

336. Müller, H., et al., *Biatrial anatomical reverse remodelling after radiofrequency catheter ablation for atrial fibrillation:: evidence from real-time three-dimensional echocardiography.* Europace, 2008. **10**(9): p. 1073-1078. （The reason for exclusion: duplicate record）

337. Müller, H., et al., *Biatrial anatomical reverse remodelling after radiofrequency catheter ablation for atrial fibrillation: Evidence from real-time three-dimensional echocardiography.* Europace, 2008. **10**(9): p. 1073-1078. （The reason for exclusion: duplicate record）

338. Marsan, N.A., et al., *Comparison of left atrial volumes and function by real-time three-dimensional echocardiography in patients having catheter ablation for atrial fibrillation with persistence of sinus rhythm versus recurrent atrial fibrillation three months later.* American Journal of Cardiology, 2008. **102**(7): p. 847-853. （The reason for exclusion: Irrelevant study outcome）

339. Efremidis, M., et al., *Ablation of atrial fibrillation in patients with heart failure: reversal of atrial and ventricular remodelling.* Hellenic J Cardiol, 2008. **49**(1): p. 19-25. （The reason for exclusion: Irrelevant study outcome）

340. Delgado, V., et al., *Fate of left atrial function as determined by real-time three-dimensional echocardiography study after radiofrequency catheter ablation for the treatment of atrial fibrillation.* American Journal of Cardiology, 2008. **101**(9): p. 1285-1290. （The reason for exclusion: Irrelevant study outcome）

341. 周风华, et al., *声学定量方法动态评价房颤时右心房结构和功能的研究 %J 山东大学学报(医学版).* 2007(09): p. 865-867+885. （The reason for exclusion: Irrelevant study outcome）

342. 张平洋, et al., *缬沙坦对非瓣膜性心房颤动患者心房重构影响的研究 %J 临床荟萃.* 2007(01): p. 49-50. （The reason for exclusion: Irrelevant study outcome）

343. 白晨, *心房颤动右心房结构重构检测方法、机制和药物干预的研究*. 2007. （The reason for exclusion: Irrelevant study outcome）

344. Kojodjojo, P., et al., *Characterization of the electroanatomical substrate in human atrial fibrillation: the relationship between changes in atrial volume, refractoriness, wavefront propagation velocities, and AF burden.* J Cardiovasc Electrophysiol, 2007. **18**(3): p. 269-75. （The reason for exclusion: Irrelevant study outcome）

345. Gaita, F., et al., *Usefulness and safety of transcatheter ablation of atrial fibrillation in patients with hypertrophic cardiomyopathy.* American Journal of Cardiology, 2007. **99**(11): p. 1575-1581. （The reason for exclusion: Irrelevant study outcome）

346. Tops, L.F., et al., *Effect of radiofrequency catheter ablation for atrial fibrillation on left atrial cavity size.* American Journal of Cardiology, 2006. **97**(8): p. 1220-1222. （The reason for exclusion: Irrelevant study outcome）

347. Tsao, H.M., et al., *Morphologic remodeling of pulmonary veins and left atrium after catheter ablation of atrial fibrillation: insight from long-term follow-up of three-dimensional magnetic resonance imaging.* Journal of cardiovascular electrophysiology, 2005. **16**(1): p. 7‐12. （The reason for exclusion: Irrelevant study outcome）

348. Tsao, H.M., et al., *Morphologic remodeling of pulmonary veins and left atrium after catheter ablation of atrial fibrillation: insight from long-term follow-up of three-dimensional magnetic resonance imaging.* J Cardiovasc Electrophysiol, 2005. **16**(1): p. 7-12. （The reason for exclusion: duplicate record）

349. Tsao, H.M., et al., *Morphologic remodeling of pulmonary veins and left atrium after catheter ablation of atrial fibrillation: Insight from long-term follow-up of three-dimensional magnetic resonance imaging.* Journal of Cardiovascular Electrophysiology, 2005. **16**(1): p. 7-12. （The reason for exclusion: duplicate record）

350. Tsao, H.M., et al., *Morphologic remodeling of pulmonary veins and left atrium after catheter ablation of atrial fibrillation: Insight from long-term follow-up of three-dimensional magnetic resonance imaging.* Journal of Cardiovascular Electrophysiology, 2005. **16**(1): p. 7-12. （The reason for exclusion: duplicate record）

351. Nct, *Cavotricuspid Isthmusblock and Circumferential Pulmonary Vein Isolation in Patients With Atrial Fibrillation.* https://clinicaltrials.gov/show/NCT00247780, 2005. （The reason for exclusion: Irrelevant study outcome）

352. Fayad, G., et al., *Endocardial radiofrequency ablation during mitral valve surgery: effect on cardiac rhythm, atrial size, and function.* Ann Thorac Surg, 2005. **79**(5): p. 1505-11. （The reason for exclusion: Irrelevant study outcome）

353. Fayad, G., et al., *Endocardial Radiofrequency ablation during mitral valve surgery: Effect on cardiac rhythm, atrial size, and function.* Annals of Thoracic Surgery, 2005. **79**(5): p. 1505-1511. （The reason for exclusion: duplicate record）

354. Fayad, G., et al., *Endocardial radiofrequency ablation during mitral valve surgery: Effect on cardiac rhythm, atrial size, and function.* Annals of Thoracic Surgery, 2005. **79**(5): p. 1505-1511. （The reason for exclusion: duplicate record）

355. Bunch, T.J., et al., *Outcomes after cardiac perforation during radiofrequency ablation of the atrium.* Journal of Cardiovascular Electrophysiology, 2005. **16**(11): p. 1172-1179. （The reason for exclusion: Irrelevant study outcome）

356. 杨振文, *应用Amplatzer封堵器治疗房间隔缺损及相关研究*. 2004. （The reason for exclusion: Irrelevant study outcome）

357. Chiappini, B., et al., *The role of corticosteroid therapy following surgery for atrial fibrillation.* J Card Surg, 2004. **19**(3): p. 232-4. （The reason for exclusion: Irrelevant study outcome）

358. Saliba, W., et al., *Circumferential ultrasound ablation for pulmonary vein isolation: analysis of acute and chronic failures.* J Cardiovasc Electrophysiol, 2002. **13**(10): p. 957-61. （The reason for exclusion: Irrelevant study outcome）

359. Gorenek, B., et al., *Immediate recurrence of atrial fibrillation after internal cardioversion: importance of right atrial conduction variations.* J Electrocardiol, 2002. **35**(4): p. 313-20. （The reason for exclusion: Irrelevant study outcome）

360. Blommaert, D., et al., *Limited internal shocks for atrial fibrillation refractory to external cardioversion.* Int J Cardiol, 1999. **71**(1): p. 71-8. （The reason for exclusion: Irrelevant study outcome）

361. Ausma, J., et al., *Structural changes of atrial myocardium due to sustained atrial fibrillation in the goat.* Circulation, 1997. **96**(9): p. 3157-3163. （The reason for exclusion: animal experiment）

362. 张云霞, 任., *纵隔占位性病变致房性心律失常三例 %J 中国循环杂志.* 1996(07): p. 66. （The reason for exclusion: case report）
