# Supplementary material for: Right atrial volume index and right atrial volume predict atrial fibrillation recurrence: A meta-analysis
Source: PLoS One. 2024 Dec 16;19(12):e0315590. doi: 10.1371/journal.pone.0315590 (PMC11649108; doi:10.1371/journal.pone.0315590)
Supplement: S9 Table — (DOCX) [file pone.0315590.s009.docx]

| **Study** | **Selectiion** | | | | **Comparability** | | **Outcome** | | | **Total** |  |
| --- | --- | --- | --- | --- | --- | --- | --- | --- | --- | --- | --- |
|  |  |  |  |  |  |  |  |  |  |  |  |
|  | **Representativeness of the exposed cohort** | **Selection of the non-exposed cohort** | **Ascertainment of exposure** | **Demonstration that outcome of interest was not present at start of study** | **Control for age and sex** | **control for other confounders** | **Assessment of outcome** | **Was follow-up long enough for outcomes to occur** | **Adequacy of follow-up of cohorts** |  |  |
|  |  |  |  |  |  |  |  |  |  |  |  |
| Mărgulescu AD | 1 | 1 | 1 | 1 | 0 | 0 | 1 | 1 | 1 | 7 |  |
| Pan T | 1 | 1 | 1 | 0 | 1 | 1 | 1 | 1 | 1 | 8 |  |
| Luong C | 1 | 1 | 1 | 1 | 1 | 1 | 1 | 1 | 1 | 9 |  |
| Moon J (2015) | 1 | 1 | 1 | 1 | 0 | 0 | 1 | 1 | 1 | 7 |  |
| Moon J (2013) | 1 | 1 | 1 | 1 | 1 | 1 | 1 | 1 | 1 | 9 |  |
| Moon J (2012) | 1 | 1 | 1 | 1 | 1 | 1 | 1 | 1 | 1 | 9 |  |
| Tomaselli M | 1 | 1 | 1 | 0 | 0 | 0 | 1 | 1 | 1 | 6 |  |
| Gunturiz-Beltrán C | 1 | 1 | 1 | 1 | 0 | 0 | 1 | 1 | 1 | 7 |  |
| Takagi T | 1 | 1 | 1 | 1 | 1 | 1 | 1 | 1 | 1 | 9 |  |
| Kumagai Y | 1 | 1 | 1 | 1 | 1 | 1 | 1 | 1 | 1 | 9 |  |
| Zhao L | 1 | 1 | 1 | 0 | 1 | 1 | 1 | 1 | 1 | 8 |  |
| Akutsu Y | 1 | 1 | 1 | 1 | 1 | 1 | 1 | 1 | 1 | 9 |  |

**S9 Table.** Study quality evaluation via the Newcastle-Ottawa Scale.
